# Engineered Bacteria‐Vesicle Delivered Lactate Reprogramming Boosts Tumor Radiosensitivity

**DOI:** 10.1002/advs.202524303

**Published:** 2026-01-04

**Authors:** Fei Peng, Zhe Lei, Zhehao Zhang, Zhiyue Su, Chonghai Zhang, Huan Yang, Shu Liu, Mengyuan Hu, Yuhong Wang, Lingchuan Guo, Lin Hu, Kai Yang

**Affiliations:** ^1^ Department of Pathology State Key Laboratory of Radiation Medicine and Protection School of Radiation Medicine and Protection & School for Radiological and Interdisciplinary Sciences (RAD‐X) the First Affiliated Hospital Collaborative Innovation Center of Radiation Medicine of Jiangsu Higher Education Institutions Cancer Institute Suzhou Medical College Soochow University Suzhou Jiangsu China

**Keywords:** engineered bacteria, lactate reprogramming, radioresistance, targeted delivery, tumor microenvironment

## Abstract

Radiotherapy (RT) remains a cornerstone in cancer treatment, yet its efficacy is often compromised by tumor‐acquired radioresistance, driven in part by lactate accumulation in the tumor microenvironment (TME). Lactate fosters therapeutic resistance through aberrant DNA repair, immunosuppression, and metabolic reprogramming, posing a formidable clinical challenge. Here, we report a precision microbial therapy leveraging engineered *Escherichia coli* Nissle 1917 (EcNΔ*nlpI* ^IHCL^, ENHL) to target and deplete lactate in the TME. By utilizing engineered bacteria with *nlpI* gene deletion to enhance outer membrane vesicles (OMVs) biogenesis and introducing a bifunctional surface display system (INP‐HlpA for tumor targeting and ClyA‐EGFP for tracking), ENHL delivers lactate oxidase (LOx) to neutralize acidic stress. In vitro and in vivo studies confirm that ENHL and LOx‐loaded OMVs effectively radiosensitize colorectal cancer cells by depleting tumor‐derived and radiation‐induced lactate. Oral administration of ENHL selectively colonizes tumors, where arabinose induction triggers localized LOx expression, significantly improving radiosensitivity and immune cell infiltration while modulating gut microbiota. This synergistic approach—combining targeted metabolic modulation with microbial precision therapy—represents a transformative strategy to overcome RT resistance in colorectal cancer, offering a promising pathway toward clinical translation.

## Graphical abstract

We engineered a bacterial‐vesicular dual‐delivery platform that targets LOx to colorectal tumors, enabling lactate clearance, immune microenvironment remodeling, and microbiota modulation. This microbe–metabolism synergistic strategy effectively sensitizes colorectal cancer to radiotherapy, offering a promising approach to overcome radioresistance.

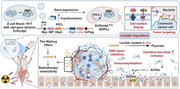

## 1 Introduction

Radiotherapy (RT) stands as a cornerstone in cancer therapy, inducing tumor cell death through DNA damage and structural disruption [1, 2]. In diseases like rectal adenocarcinoma, its role is amplified within multimodal strategies, offering well‐established benefits in both neoadjuvant and adjuvant settings [3, 4]. However, a significant clinical hurdle remains: the emergence of acquired radioresistance, driven in part by the tumor microenvironment (TME) [5, 6]. Radiation induces profound metabolic reprogramming, upregulating glycolytic enzymes and exacerbating hypoxia, which fosters a reliance on glycolysis and leads to the accumulation of lactate within the TME [7, 8]. The abundant lactate in microenvironment acts as a multifunctional signaling molecule, actively promoting radioresistance through aberrant DNA repair, apoptosis inhibition, metabolic reprogramming, and the establishment of an immunosuppressive microenvironment [9, 10]. Moreover, subsequent investigations have revealed a strong correlation between radioresistance and lactate accumulation within tumors [11, 12]. Accordingly, it is an effective strategy to overcome resistance to radiotherapy by modulating lactate levels within the tumor microenvironment. In this context, lactate oxidase (LOx) has emerged as a promising biocatalytic tool in anticancer therapy by virtue of its unique enzymatic capacity to selectively catalyze lactate conversion into pyruvate [13, 14, 15]. To date, various nanocarriers have been employed for LOx delivery, including polymeric nanoparticles, nanozymes, hydrogels, and inorganic nanomaterials, which can degrade lactate in the TME, effectively reversing immunosuppression and demonstrating promising potential in cancer therapy [14, 15, 16]. However, existing nanocarriers often suffer from limitations such as poor biocompatibility, inadequate tumor tissue penetration, and non‐degradability, which constrain their clinical translational potential [14]. Therefore, delivery systems with excellent biocompatibility and tissue‐penetrating capabilities—such as engineered microbes, cell membranes and their derivatives, and exosomes—are emerging as promising new strategies for LOx delivery [14, 17].

Engineered bacteria, particularly probiotic strains like *Escherichia coli* Nissle 1917 (EcN), offer a promising avenue for targeted therapy due to their inherent tumor tropism and, driven by the hypoxia of TME or chemotactic motility [18, 19, 20, 21, 22]. EcN, owing to its well‐established excellent biosafety and high protein expression capacity, serves as an ideal vector for tumor‐targeted therapeutic delivery, providing key advantages for its clinical translation [23]. A key innovation lies in leveraging the bacteria's outer membrane vesicles (OMVs), nanoscale (20–200 nm) nanoparticles naturally secreted by Gram‐negative bacteria [24, 25]. These OMVs possess intrinsic properties ideal for nanomedicine: they can penetrate biological barriers, exhibit efficient tumor targeting, and serve as natural delivery vehicles [26, 27]. Moreover, the *nlpI* knockout strain (EcNΔ*nlpI*) lacked a key bacterial outer membrane lipoprotein and increased OMVs production without impairing membrane integrity [27, 28]. Efficient surface display of therapeutic payloads has been achieved using ice nucleation protein (INP) and cytolysin A (ClyA) domains [29, 30, 31]. Therefore, a critical strategy toward oral protein delivery using engineered bacteria is to ensure that surface‐displayed proteins remain stable in the gastrointestinal tract, enabling them to reach tumor sites, establish colonization, and penetrate tumor tissues to effectively regulate tumor metabolism.

**FIGURE 1:**
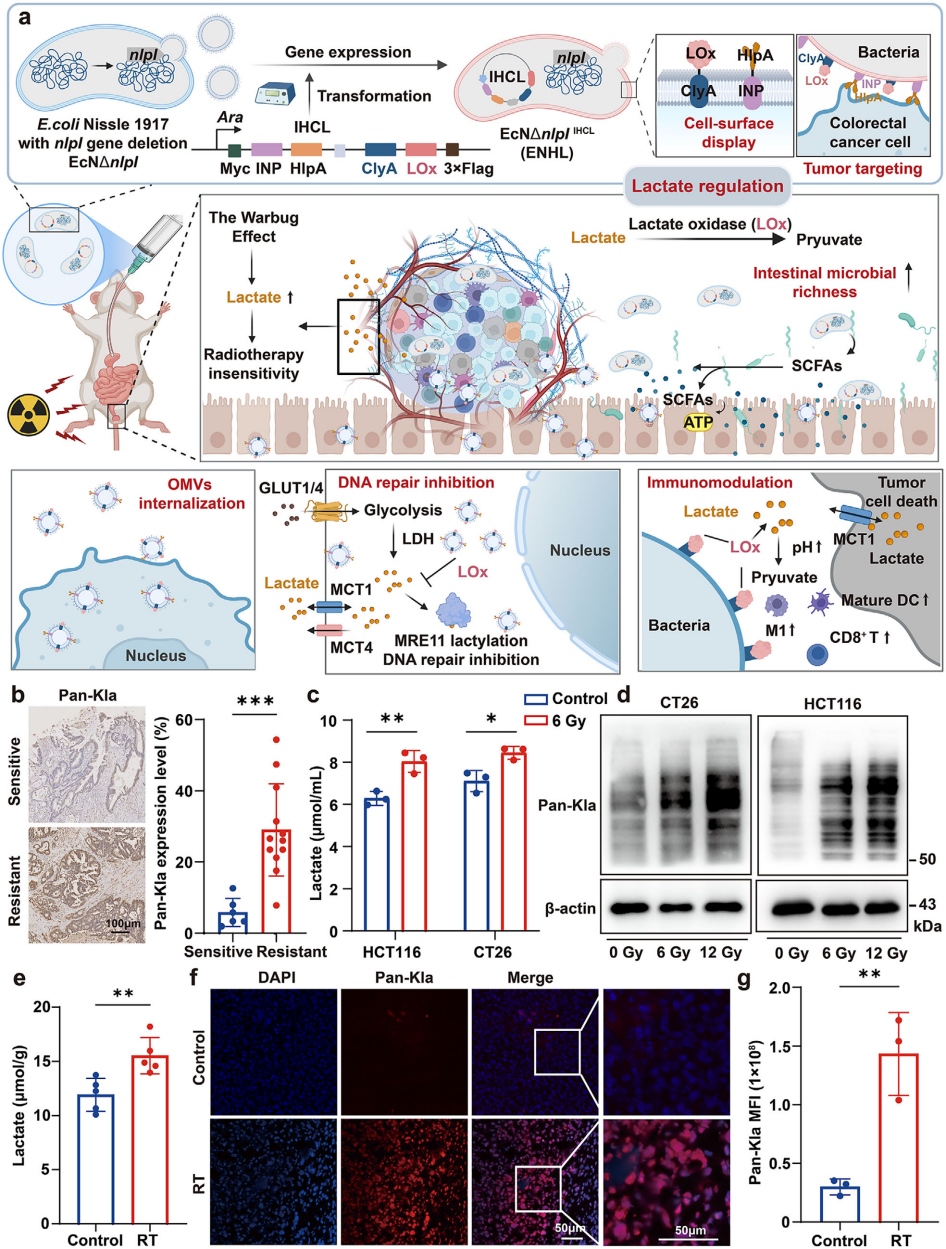
Radiotherapy upregulated tumor lactate and lactylation. (a) Engineered bacteria targeting tumor lactate metabolism: a novel approach for modulating the colorectal cancer microenvironment. Created with BioRender. (b) Representative images (left) and quantitative analysis (right) of Pan‐Kla staining in Resistant and Sensitive patient tissues (The cohort included 6 radio‐chemotherapy‐sensitive cases and 12 resistant cases.); scale bars: 100 µm. (c) Lactate concentrations in culture supernatants of CT26 and HCT116 cells following 6 Gy irradiation. (d) Western blot analysis of pan‐lysine lactylation (pan‐Kla) modification levels in whole protein extracts from irradiated CT26 and HCT116 cells. (e) Lactate levels in CT26 xenograft tumors from mice treated with or without fractionated irradiation (RT; 6 Gy/fraction × 3 fractions, administered every 2 days; *n* = 5). (f,g) Representative immunofluorescence images of Pan‐Kla (red) in CT26 xenografts (left; scale bar: 50 µm), along with quantified mean fluorescence intensity (MFI) of Cy3 channel within Pan‐Kla‐positive regions of interest (right; *n* = 3). Data were presented as mean ± SD. Statistical significance was assessed by unpaired two‐tailed Student's *t*‐test (***p < 0.001, **p < 0.01, *p < 0.05).

Building on these principles, we engineered the EcNΔ*nlpI* strain with a bifunctional surface display system comprising a targeting module (INP‐histone‐like protein A, HlpA) and a reporting module (ClyA‐EGFP), allowing both specific tumor targeting and membrane‐localized fluorescent tracking. To address TME acidification, we constructed the EcNΔ*nlpI* ^IHCL^ (ENHL) strain to express a ClyA‐LOx fusion protein, incorporating LOx as a key biocatalytic component for neutralizing lactate‐induced acidity. Next, we confirmed that these LOx‐displaying OMVs effectively penetrate the intestinal epithelial barrier and enter the cells, neutralize lactate, and thereby sensitize colorectal cancer (CRC) cells to ionizing radiation. Upon oral administration, the engineered strain, ENHL, selectively colonized colorectal tumors, which subsequent induction with arabinose triggered localized expression of the therapeutic protein. The results demonstrated that ENHL significantly enhanced radiosensitivity and improved immune cell infiltration in CRC tumors by LOx‐mediated depletion of both tumor‐derived and radiation‐induced lactate, while concurrently modulating the intestinal microbiota (Figure 1a). More importantly, this study synergistically combined targeted metabolic modulation with precision microbial therapy, providing a clinical transformation approach to overcome therapeutic resistance in CRC.

## 2 Results

### 2.1 RT Upregulated Tumor Lactate and Lactylation

According to literatures, lactate enhances the DNA damage repair capacity of tumor cells by promoting the lactylation of key DNA repair proteins, such as MRE11 and NBS1 [32, 33], and also modulates chromatin architecture through histone lysine lactylation (Kla) modifications [34, 35, 36]. Therefore, this lactate‐enriched microenvironment facilitates the development of tumor radioresistance through a multilayered regulatory network [37]. To assess the relationship between lactylation and radioresistance, we profiled pan‐lysine lactylation (Pan‐Kla) expression in 18 colorectal cancer specimens. The cohort comprised 6 cases sensitive to neoadjuvant radio‐chemotherapy and 12 cases resistant to neoadjuvant radio‐chemotherapy (Table S1). The results showed that higher lactylation levels were observed in the resistant group compared to sensitive cases (*p* < 0.001) (Figure 1b). Lactate functions as the essential metabolic substrate for protein lactylation, facilitating post‐translational modifications in histone and non‐histone proteins [38, 39]. Moreover, radiation‐induced vascular damage exacerbates hypoxia within the TME, and this metabolic stress drives malignant cells to rely more heavily on glycolysis for energy production [40, 41]. Under hypoxic conditions, the activation of hypoxia‐inducible factor‐1alpha (HIF1α) further boosts glycolytic activity, leading to the accumulation of lactate in the TME [42, 43, 44]. Moreover, existing evidence indicates that irradiation upregulates LDHA protein expression [12, 45]. Therefore, we analyzed the expression patterns of HIF1α and lactate dehydrogenase A (LDHA) in 18 colorectal cancer specimens through systematic immunofluorescence staining. It was found that the resistant group exhibited higher protein levels of both HIF1α and LDHA than those of sensitive cases (Figure S1). Next, we also analyzed 594 colorectal adenocarcinoma samples from TCGA PanCancer Atlas and found a significant positive correlation between LDHA mRNA expression and Winter hypoxia scores (r = 0.4212, *p* < 0.0001, Figure S2). Collectively, these findings suggest that radioresistance is closely associated with lactylation and lactate metabolism.

To further investigate radiation‐induced lactate metabolic alterations, we performed in vitro X‐ray irradiation experiments on two colorectal cancer cell lines, CT26 and HCT116. The results showed that, compared to the control group, lactate concentrations in the cell culture supernatants significantly increased 24 h after exposure to 6 Gy (Figure 1c). Then, we further revealed a radiation dose‐dependent increase in protein lactylation via western blot through using pan‐Kla antibody (Figure 1d). Similarly, we found that tumor tissues subjected to 6 Gy×3 fractionated X‐ray irradiation showed significant increases in both lactate levels and Pan‐Kla modification compared to the non‐irradiated group in the CT26 tumor‐bearing mouse model (Figure 1e–g and Figure S3). Importantly, intracellular lactate had been shown to promote lactylation modifications of various proteins which promote drug resistance [46]. Consistently, we found that lactate exposure markedly enhanced cellular DNA repair capacity through comet assays, thereby contributing to radioresistance (Figure S4a–c). These findings suggest that therapeutic targeting of the lactate metabolic network—including its production, transport, and degradation pathways—can represent a promising strategy to overcome the radioresistance in colorectal cancer.

### 2.2 Construction and Activity Validation of Tumor‐Targeting Engineered Bacteria

**FIGURE 2:**
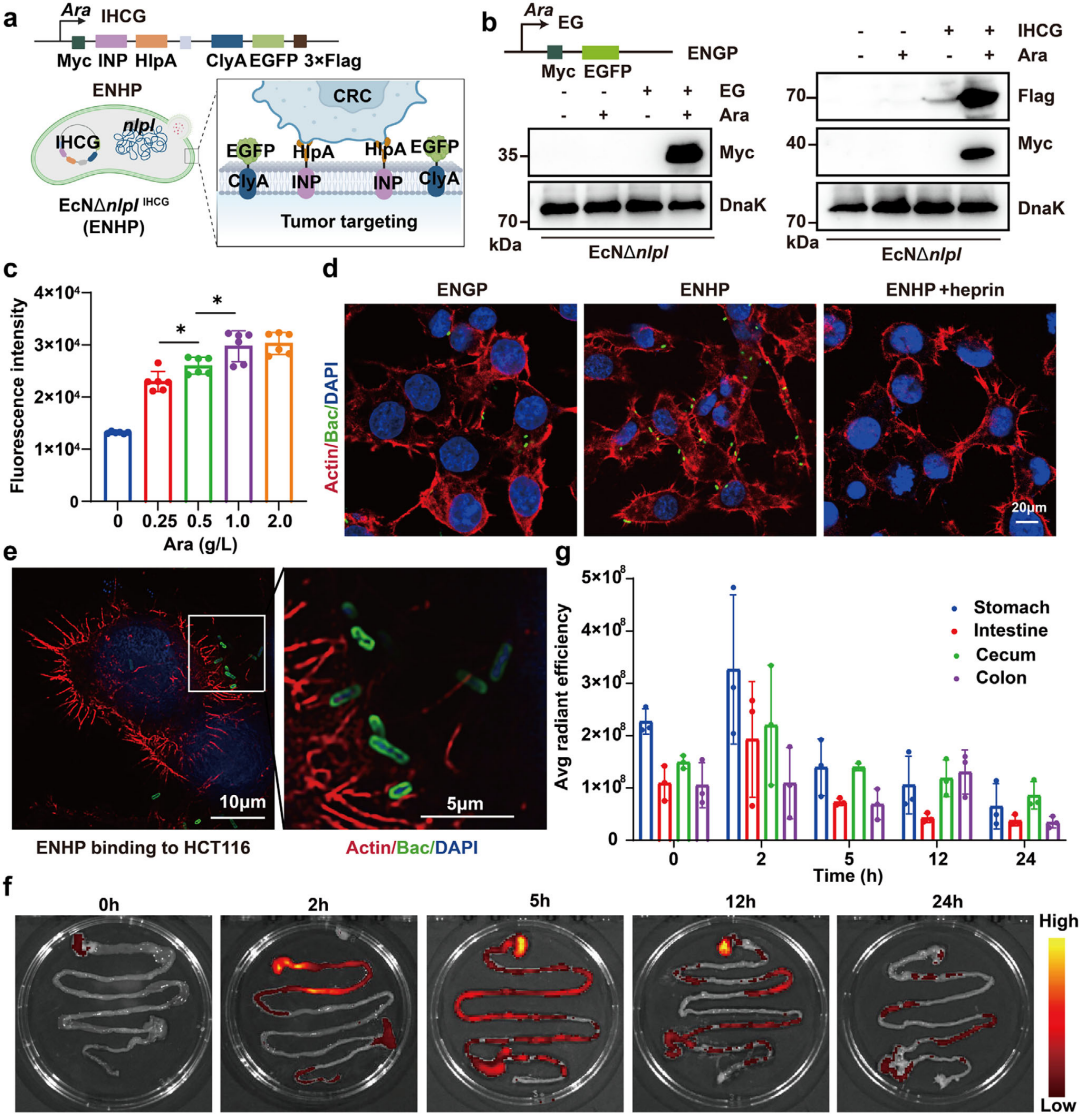
The construction of tumor‐targeting engineered bacteria. (a) The schematic of engineered EcN is designed for construction and tumor targeting. Created with BioRender. (b) Western blot verification of Flag/Myc‐tagged protein expression in engineered bacteria under Ara induction. (c) Optimization of Ara induction concentrations for maximal protein expression in vitro (*n* = 6). (d) Representative fluorescence images demonstrating bacterial binding to host cells. Bacteria (green; EGFP labeled), actin filaments (red; phalloidin), and nuclei (blue; DAPI); scale bar: 20 µm. (e) Super‐resolution imaging shows EGFP localization on bacterial membranes (green) and bacterial colonization on HCT116 cell surfaces, actin filaments (red), and nuclei (blue); scale bar: 10 µm. (f,g) In vivo tracking of ENHP: Representative fluorescence images (f) and quantified biodistribution (g) in gastrointestinal tissues at specified time points (*n* = 3). All data represent mean ± SD. Statistical significance was determined by one‐way ANOVA with Tukey's multiple‐comparisons test (*p < 0.05, ** *p* < 0.01).

Genetically engineered EcN and its secreted OMVs show significant potential for oral protein delivery, with OMVs effectively transporting proteins across the intestinal epithelial barrier [26, 27]. Compared to the wild‐type EcN, EcNΔ*nlpI* exhibited a significantly higher number of OMVs in LB medium (2.09 ± 0.49‐fold increase, *p* < 0.01), as quantified by bicinchoninic acid (BCA) assay, while maintaining similar growth kinetics (Figures S5 and S7). Next, EcNΔ*nlpI* was transformed with pBAD‐His‐based plasmids carrying arabinose‐inducible promoters. The engineered strain EcNΔ*nlpI* ^IHCG^ (ENHP) was constructed by transforming EcNΔ*nlpI* with the pBAD‐His‐Myc‐INP‐HlpA‐RBS‐ClyA‐EGFP‐3×Flag (IHCG) plasmid, for co‐expression of fusion proteins of INP‐HlpA and ClyA‐EGFP. Similarly, the plasmid pBAD‐His‐Myc‐EGFP (EG) was transformed into EcNΔ*nlpI* to generate the strain designated as EcNΔ*nlpI* ^EG^ (ENGP), for lacking HlpA and EGFP expression alone. All the plasmid information was in Figure S6 and Table S2. Through the western blot analysis, we confirmed the successful plasmid expression in both engineered strains and their OMVs (Figure 2a,b and Figure S8). Under arabinose induction, ENHP expressed Myc‐ and 3×Flag‐tagged proteins, respectively, whereas ENGP expressed only the Myc‐tagged protein (Figure 2b). Moreover, we screened the induction conditions using the EGFP fluorescent reporter system and found that the fluorescence intensity reached a peak at an induction concentration of 1 g/L arabinose (Ara) (Figure 2c). Therefore, we selected the arabinose concentration of 1 g/L for subsequent in vitro induction experiments to achieve optimal protein expression.

To evaluate the tumor‐targeting capability of the engineered strains, we first characterized heparin sulfate proteoglycan (HSPG) expression patterns in various tumor cell lines. The results showed that HSPG was highly expressed on CT26 and HCT‐116 cells (Figure S9). Subsequently, ENHP and ENGP were co‐incubated with CT26 cancer cells, respectively, and flow cytometry analysis revealed a significantly higher bacterial binding rate in the ENHP group compared to the ENGP control group (Figure S10). Additionally, we also visualized the binding of bacteria to tumor cells by confocal laser scanning microscopy (CLSM), and the results showed that the binding of ENHP to CT26 and HCT‐116 cells was significantly higher than that of ENGP (Figure 2d and Figure S11). However, this enhanced binding was effectively blocked by heparin pretreatment (Figure S12). Notably, it was revealed that a distinct ring‐like distribution of EGFP distribution on bacterial membranes by super‐resolution microscopy (Figure 2e), indicating the presence of recombinant proteins on bacterial OMVs. To investigate the in vivo behavior of engineered bacteria within the complex physiological environment, we performed serial in vivo imaging system (IVIS) analysis to characterize the spatiotemporal distribution profile of engineered bacteria in the intestinal tract at predetermined points (0, 2, 5, 12, and 24 h post‐administration) after oral gavage administration of 1 × 10^9^ CFU ENHP with 30 g/Lc arabinose solution. Then, the results showed that the bioluminescent signal was predominantly localized in the cecum 2 h after oral gavage and subsequently migrated to the colonic region by 12 h (Figure 2f,g). These findings provided direct visual evidence for the surface display of recombinant proteins and HlpA‐mediated targeting of our engineered bacterial system to colon cancer cells.

**FIGURE 3:**
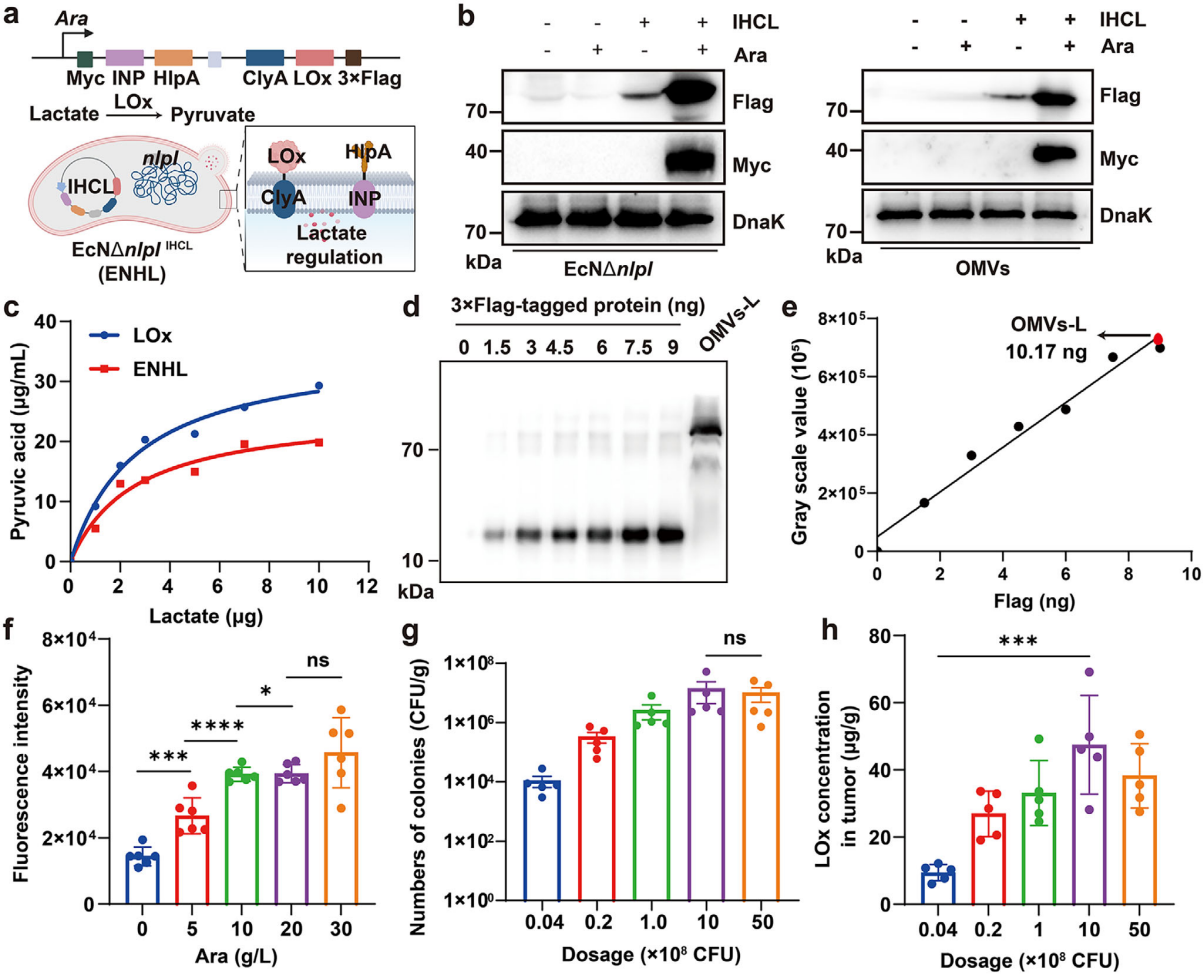
LOx activity exhibited by engineered bacteria. (a) Schematic of LOx‐expressing engineered bacteria and their functional mechanism. Created with BioRender. (b) Western blot analysis of 3×Flag‐/Myc‐tagged protein expression in engineered bacteria and derived OMVs. (c) Comparative enzyme kinetics analysis of pyruvate generation between native LOx and ENHL. (d,e) Western blot detection (d) and quantitative grayscale analysis (e) of Flag‐tagged proteins in purified OMVs. (f) In vivo optimization of arabinose induction concentration (*n* = 5). (g) Tumor colonization efficiency of ENHL in CT‐26 orthotopic tumor models at different bacterial doses (*n* = 5). (h) Tumor LOx concentration measured by Flag‐ELISA after ENHL administration (*n* = 5). All data represent mean ± SD. Statistical significance was determined by one‐way ANOVA with Tukey's multiple‐comparisons test (*p < 0.05, ** *p* < 0.01, *** *p* < 0.001, **** *p* < 0.0001, ns. not significant).

Building upon our previously established outer membrane‐anchored expression system with validated capacity for efficient target protein production, we supposed that LOx‐mediated lactate degradation could address lactate accumulation‐induced microenvironment acidification, immunosuppression, and therapeutic resistance in the TME. Accordingly, we innovatively integrated the *LOX* gene into this system to precisely regulate intratumoral lactate levels. The engineered strain EcNΔ*nlpI* ^IHCL^ (ENHL) was constructed by transforming EcNΔ*nlpI* with the pBAD‐His‐Myc‐INP‐HlpA‐RBS‐ClyA‐LOx‐3×Flag (IHCL) plasmid (Figure 3a). As shown in Figure 3b, using western blot analysis, the Myc‐ and 3×Flag‐tagged proteins expression was detected both in bacterial cells and their secreted OMVs (OMVs‐L) after Ara induction. To investigate whether antibiotic exposure affects LOx expression, ENHL bacteria were cultured in LB medium with or without ampicillin, followed by immunoblotting using a Flag tag. The results showed that ampicillin had no significant effect on LOx expression (Figure S13). Afterwards, enzymatic characterization revealed that, compared to the LOx standard, lactate oxidase expressed by the engineered strain ENHL showed a slight decrease in substrate affinity, with its Michaelis constant (Km) increasing from 2.61 to 2.69 mm, while retaining some native enzymatic activity (Figure 3c). To quantify LOx loading on OMVs, we performed Flag‐tagged proteins as a proxy for LOx content via Western blot analysis, which demonstrated an average Flag loading of 10.17 ng per 10 µg of OMVs‐L (Figure 3d,e). Moreover, we performed the LOx‐specific enzyme‐linked immunosorbent assay (ELISA) to precisely quantify LOx protein loading on engineered bacteria and their OMVs. The analysis revealed LOx concentrations of 180.96 ± 7.01 ng in 1.0 × 10^8^ CFU of bacterial suspension and 125.87942 ± 10.05 ng in 10 µg of OMVs‐L (Figure S14).

To determine the optimal arabinose concentration for in vivo induction while minimizing gastrointestinal side effects, we administered mice with 1 × 10⁹ CFU of engineered bacteria via oral gavage, followed by a 12‐hour induction with varying arabinose concentrations. And then, fecal samples were collected in PBS (1 mL/g feces) and analyzed for fluorescence intensity. The results showed that maximum induction was achieved at 10 g/L arabinose (Figure 3f). Additionally, we found that the engineered bacteria exhibited dose‐proportional tumor colonization, achieving maximum load capacity at 1.0 × 10⁹ CFU (Figure 3g), suggesting a saturation threshold for bacterial delivery. Consistently, the actual protein production of bacteria in the tumor was basically the same as the upward trend of the bacterial load in the tumor after oral administration of different doses of bacteria (Figure 3h). While higher oral doses were technically feasible, we observed dose‐limiting diarrhea and behavioral depression in mice. Therefore, 1 × 10⁹ CFU was selected as the optimal therapeutic dose for subsequent experiments. Finally, we demonstrated efficient tumor homing of orally delivered engineered bacteria, which preferentially accumulated in the stromal compartment by confocal microscopy tracking (Figure S15). Importantly, bacterially secreted Lox was widely distributed throughout the tumor stroma, demonstrating a spatial pattern that facilitated targeted LOx delivery and effective in situ lactate catabolism.

### 2.3 LOx‐Loaded OMVs Improved Radiosensitivity Through Lactate Regulation

**FIGURE 4:**
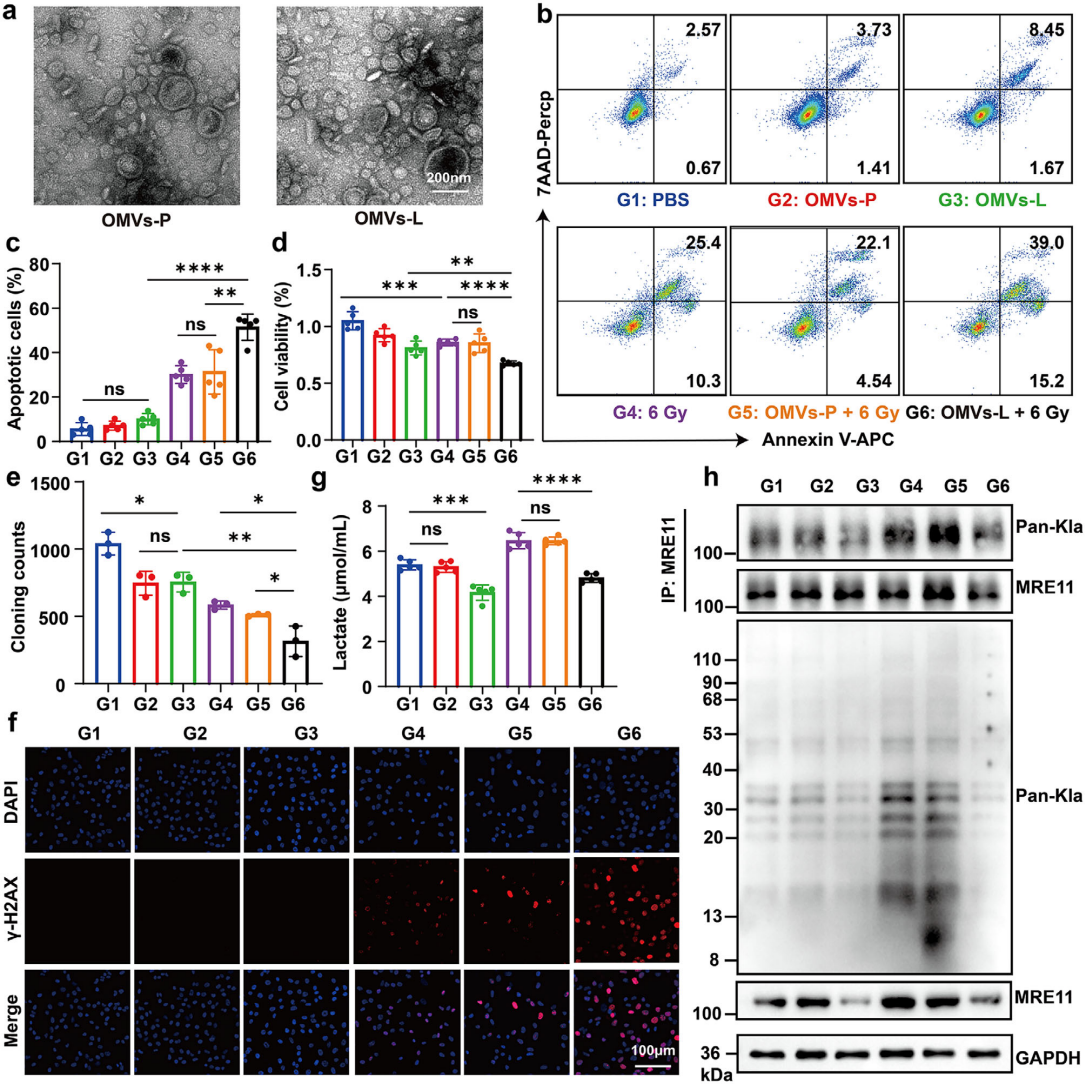
In vitro cytotoxicity and mechanistic analysis of LOx‐loaded OMVs. (a) TEM characterization of OMVs‐P (OMVs derived from ENHP) and OMVs‐L (OMVs derived from ENHL) morphology; scale bar: 200 nm. (b,c) Flow cytometric analysis of CT26 cell apoptosis: representative plots (b) and quantitative analysis (c) following treatments (G1: PBS control; G2: OMVs‐P; G3: OMVs‐L; G4: 6 Gy; G5: OMVs‐P + 6 Gy; G6: OMVs‐L + 6 Gy; *n* = 5). (d) Cell viability assessment of CT26 cells post‐treatment (*n* = 5). (e) Quantification of colony counts with different treatments towards CT26 cells. (f) Immunofluorescence images of γ‐H2AX staining after different treatments; scale bar: 100 µm. (g) The lactate content in the supernatant of CT26 cells after different treatments (*n* = 5). (h) Western blot analysis of CT26 cell lysates for Pan‐Kla, GAPDH, and MRE11 expression, along with Pan‐Kla‐modified MRE11 levels under different treatments. All data represent mean ± SD. Statistical significance was determined by one‐way ANOVA with Tukey's multiple‐comparisons test (*p < 0.05, ** *p* < 0.01, *** *p* < 0.001, **** *p* < 0.0001, ns. not significant).

Building on the confirmation that ENHL could efficiently secrete LOx‐loaded therapeutic OMVs (OMVs‐L), we conducted a comprehensive investigation into the radiosensitization effects of LOx‐loaded OMVs. Firstly, OMVs were isolated by ultracentrifugation and characterized using transmission electron microscopy (TEM), which revealed that all OMVs exhibited a typical bilayered membrane structure (Figure 4a). The particle size distributions of ENHP‐derived OMVs (OMVs‐P) and ENHL‐derived OMVs (OMVs‐L) were around 100–200 nm (Figure S16). The zeta potential values of EcNΔ*nlpI*‐derived OMVs (OMVs), OMVs‐P, and OMVs‐L were −15.27 ± 0.57 mV, −21.60 ± 5.21 mV, and −21.53 ± 1.301 mV, respectively (Figure S17). The surface potentials of OMVs, OMVs‐P, and OMVs‐L were lower than those of unloaded OMVs, indicating that the protein was successfully modified on the surface of OMVs. Next, we assessed the cellular internalization capacity of these bacterial OMVs, revealing that OMVs‐P exhibited significantly enhanced uptake in CT26 cells after 24 h of co‐incubation (Figure S18). Additionally, we found that OMVs‐P could promote the penetration of the target protein across the intestinal barrier and into intestinal tissues (Figure S19). This phenomenon suggested that OMVs might also possess the ability to penetrate tumor tissues, thereby exerting therapeutic effects within the tumor.

Then, we assessed the viability of CT‐26 cells treated with varying concentrations of OMVs‐P and OMVs‐L using CCK‐8 assays after 24 h incubation, revealing that OMVs (≤100 µg/mL) alone maintained a cell survival rate >50% (Figure S20). Subsequently, we evaluated the cytotoxicity of different formulations (including PBS (G1), OMVs‐P (G2), OMVs‐L (G3), 6 Gy (G4), OMVs‐P + 6 Gy (G5), OMVs‐L + 6 Gy (G6)) on tumor cells. As illustrated in Figure 4b,c, flow cytometry with Annexin V‐APC/7‐AAD staining demonstrated that 100 µg/mL OMVs‐L markedly increased CT26 cell apoptosis when combined with 6 Gy radiation. Similarly, 50 µg/mL OMVs‐L showed significant radiosensitization under 6 Gy X‐ray treatment (Figure 4d). Colony formation assays further confirmed the enhanced radiation response, with OMVs‐L + 6 Gy (G6) showing significantly reduced clonogenic survival compared to OMVs‐P + 6 Gy (G5) or radiation alone(G4) (Figure 4e and Figure S21). These collective results suggested that OMVs‐L potentiated radiation‐induced cell death, supporting its role as an effective radiosensitizers.

To explore the relevant mechanism, we detected both the expression of the lactylation of DNA damage protein MRE11 and status of γH2AX, a canonical DNA double‐strand break marker. In addition, we detected the immunofluorescence staining of γH2AX foci formation and found significantly greater DNA damage accumulation in OMVs‐L + 6 Gy (G6) treated cells compared to both untreated controls and radiation monotherapy (Figure 4f and Figure S22). Next, we quantified the lactate levels in the supernatant of CT26 cells following various treatments to evaluate the functional capacity of OMVs‐L. The results demonstrated that OMVs‐L effectively degraded radiotherapy‐induced lactate of CT26 cells (Figure 4g), suggesting its capacity to remodel the tumor microenvironment through targeted lactate catabolism. To further study the effect of OMVs‐L on the lactylation of cancer cell proteins, we detected the expression level of Pan‐Kla by Western blot and immunofluorescence staining (Figure 4h and Figure S23 and S24). The results showed that OMVs‐L treatment significantly reduced the upregulated Pan‐Kla expression in CT26 cells caused by radiotherapy. Western Blot assays showed that OMVs‐L treatment significantly reduced the lactylation of MRE11 in CT26 cells (Figure 4h). These findings collectively demonstrated that LOx‐loaded OMVs exerted radiosensitizing effects through a multi‐faceted mechanism: (1) targeted lactate consumption in the tumor microenvironment, (2) suppression of protein lactylation modifications, and (3) impairment of DNA damage repair pathways. This synergistic approach significantly enhanced the anti‐tumor efficacy of radiotherapy.

### 2.4 Antitumor Abilities of ENHL‐Radiotherapy Combination In Vivo

To assess the biosafety of oral ENHL administration, mice were divided into control and ENHL‐treated groups (1 × 10^9^ CFU per dose) and received oral gavage every 2 days for one month, followed by the analysis of hematological parameters (including white blood cell count, red blood cell count, and platelet levels) and serum biochemical markers (comprising hepatic function indicators ALT and AST, as well as renal function parameters BUN and Cr). There were no statistically significant differences between ENHL‐treated and control groups, demonstrating the excellent biosafety of oral ENHL administration (Figure S25).

**FIGURE 5:**
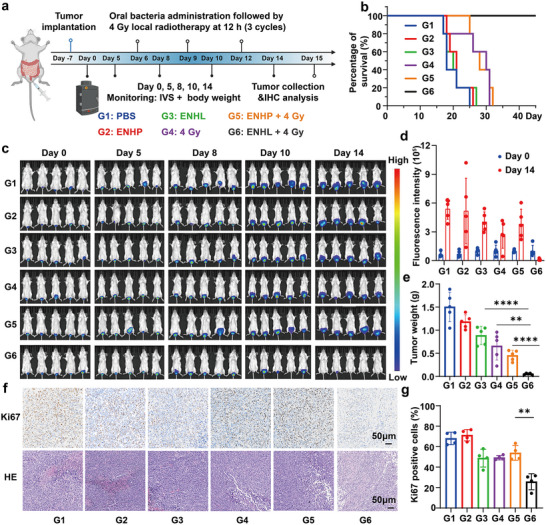
Antitumor efficacy of ENHL combined with radiotherapy. (a) Experimental design schematic for orthotopic colorectal cancer treatment. Created with BioRender. (b) Survival differences were evaluated using the log‐rank test (HR = 0.046; 95% CI, 0.007–0.32; median survival: G1 = 18 days, G6 = 53 days; *n* = 5). (c, d) Longitudinal tumor monitoring: representative bioluminescence images (c) and quantitative photon flux analysis (d) at baseline (Day 0) and endpoint (Day 14) (*n* = 5). (e) Final tumor weights of different treatment groups (*n* = 5). (f) Histological sections showing H&E staining and Ki67 immunohistochemistry; scale bar = 50 µm. (g) Quantifications of Ki67 immunohistochemistry in tumor sections. All data represent mean ± SD. Statistical significance was determined by one‐way ANOVA with Tukey's multiple‐comparisons test (*p < 0.05, ** *p* < 0.01, *** *p* < 0.001, **** *p* < 0.0001).

Given the excellent safety profile of ENHL demonstrated above, we subsequently evaluated the therapeutic potential of this strategy against conventional colorectal cancer through systematic assessment of ENHL combined with X‐ray irradiation. We firstly established the orthotopic rectal cancer model by intrarectal injection of CT26‐luc cells into anesthetized BALB/c mice at Day ‐7 (Figure 5a). Successful tumor engraftment was verified via IVIS bioluminescence imaging on Day 0. Afterwards, the mice were randomly assigned to six treatment groups and received the following treatments: PBS gavage (G1), ENHP gavage (G2), ENHL gavage (G3), 4 Gy (G4), ENHP gavage + 4 Gy (G5), ENHL gavage + 4 Gy (G6). A standardized treatment protocol was employed in which engineered bacterial suspensions (1 × 10⁹ CFU/200 µL) were administered via oral gavage, followed by a 12‐hour interval for bacterial colonization and protein expression, after which localized tumor irradiation at a dose of 4 Gy was delivered. Compared with monotherapy, the combination therapy (G6) significantly prolonged mouse survival (Figure 5b). Tumor progression was monitored by IVIS bioluminescence imaging on days 0, 5, 8, 10, and 14, demonstrating that ENHL+ 4 Gy‐treated mice (G6) exhibited significantly reduced fluorescence intensity (Figure 5c,d). This therapeutic effect was further confirmed by endpoint tumor weight measurements, with the ENHL+4 Gy group (G6) showing minimal tumor mass and size (Figure 5e and Figure S26), demonstrating that our engineered bacteria‐radiotherapy combination effectively suppressed low rectal cancer progression. Meanwhile, no significant body weight changes were observed across treatment groups (Figure S27), suggesting good treatment tolerability. In addition, the histopathological analysis of post‐treatment tumor tissues also showed that ENHL + 4 Gy group (G6) exhibited widespread necrosis/apoptosis (Figure 5f). Consistently, Ki67 IHC staining images analysis demonstrated markedly reduced tumor cell proliferation in the combination of ENHL and 4 Gy irradiation (Figure 5f,g). Finally, to comprehensively evaluate systemic toxicity, we performed H&E‐stained sections of major organs (heart, liver, spleen, lungs, and kidneys) from mice in all experimental groups. The results showed that no obvious pathological changes were observed in the ENHL‐treated group compared with the control group, indicating that ENHL had no significant toxic effects on major organs (Figure S28).

### 2.5 Molecular Mechanisms of ENHL in Enhancing Tumor Radiosensitivity

**FIGURE 6:**
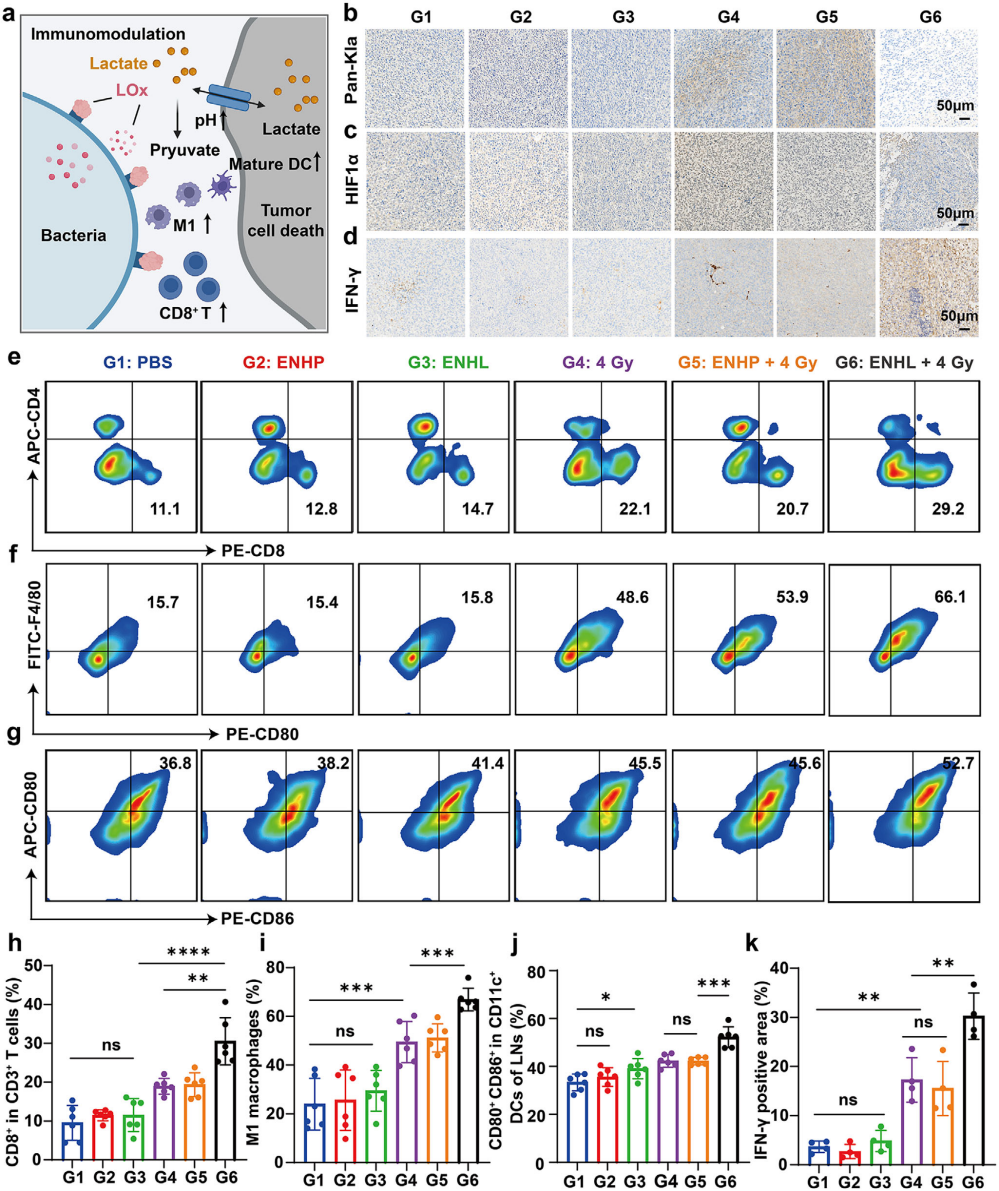
In vivo immune remodeling and tumor microenvironment modulation. (a) Schematic diagram of tumor microenvironment remodeling post‐treatment. Created with BioRender. (b–d) Representative IHC staining of Pan‐Kla, HIF1α, and IFN‐γ in tumor sections, scale bar = 50 µm. (e,f) Representative flow cytometry images of CD8^+^ T cells (CD45^+^CD3^+^CD8^+^) and M1 macrophages (CD11b^+^F4/80^+^CD86^+^) in tumor tissues. (g) Representative flow cytometry images of DCs (CD11c^+^CD80^+^CD86^+^) in mesenteric lymph nodes. (h–j) Quantitative immune cell profiling: (h) CD8^+^ T cell infiltration, (i) M1 macrophage polarization, and (j) DC maturation in mesenteric lymph nodes (*n* = 6). (k) Quantification of IFN‐γ immunohistochemical staining in mice from different treatment groups (*n* = 4). All data represent mean ± SD. Statistical significance was determined by one‐way ANOVA with Tukey's multiple‐comparisons test (*p < 0.05, ** *p* < 0.01, *** *p* < 0.001, **** *p* < 0.0001, ns. not significant).

Tumor‐derived lactate is widely recognized as a key immunosuppressive metabolite that critically impairs immune cell function within the TME [37]. We supposed a schematic diagram to illustrate how engineered bacteria, in combination with radiotherapy, thus modulating the tumor immune microenvironment (Figure 6a). Firstly, tumor tissues were collected from mice after different treatments and weighed to quantitatively assess intratumoral lactate levels. Lactate quantification revealed that oral administration of ENHL resulted in a significant reduction of lactate content within the tumor tissues (Figure S29). Second, we collected tumor tissues from mice with different treatments and performed Pan‐Kla IHC staining. The results demonstrated that LOx‐loaded ENHL (G6) markedly reduced the radiation‐induced upregulation of Pan‐Kla (G4‐5) (Figure 6b). Research demonstrates that lactate secretion exacerbates local hypoxia by reducing extracellular pH and inhibiting tumor vascular normalization [47], while inhibition of LDHA or MCT1 activity can significantly decrease intratumoral lactate levels, thereby ameliorating the hypoxic microenvironment [48, 49]. Given previous reports demonstrating hypoxia‐induced enhancement of lysine lactylation [34], we further assessed the expression levels of HIF1α in different treatment groups and observed that ENHL + 4 Gy group effectively alleviated radiation‐induced hypoxia in the TME (Figure 6c). Therefore, we guessed that the observed amelioration of intratumoral hypoxia might be attributable to lactate reduction induced by the combination therapy.

To evaluate the immunomodulatory effects of engineered bacteria in combination with radiotherapy, we analyzed tumor‐infiltrating T cells and M1‐like tumor‐associated macrophages (TAMs) using flow cytometry and IFN‐γ expression by IHC following various treatment regimens (Figure 6d–f). Compared to mice treated with PBS, those tumors of mice treated with ENHL+ 4 Gy (G6) exhibited a significant increase in the percentage of CD8^+^ T cells from 11.1% to 29.2% (Figure 6e), while the percentage of M1‐like TAMs increased significantly from 15.7% to 66.1% (Figure 6f). We further performed IHC analysis to assess IFN‐γ expression and its functional immune cell infiltration within tumor tissues. As expected, the expression level of IFN‐γ and CD8^+^ T cells were the highest in the ENHL + 4 Gy (G6) treated group (Figure 6d and Figure S30). In addition, we evaluated the antigen‐specific immune response in the mesenteric lymph nodes. The proportion of mature DC cells (CD11c^+^ CD80^+^ CD86^+^) in DCs in the 4 Gy group increased compared with the control group, and this effect was stronger in mice in the ENHL + 4 Gy (G6) group (Figure 6g). The gating strategy and representative flow cytometry data were shown in Figures S31–S33. Notably, the combination of ENHL and 4 Gy (G6) radiation therapy markedly potentiated antitumor immunity, as evidenced by enhanced CD8^+^ T cell infiltration (Figure 6h), M1‐polarized tumor‐associated macrophages (Figure 6i), increased mature dendritic cells in mesenteric lymph nodes (Figure 6j), and elevated IFN‐γ levels within the tumor microenvironment (Figure 6k). These effects might be mediated through ENHL‐mediated degradation of tumor‐derived lactate. Importantly, ENHL monotherapy also demonstrated immunomodulatory potential, as evidenced by its positive impact on mature DCs, suggesting that both its intrinsic immunogenicity and targeted lactate metabolism might collectively contribute to reprogramming the immunosuppressive TME.

### 2.6 Gut Flora Assessment

**FIGURE 7:**
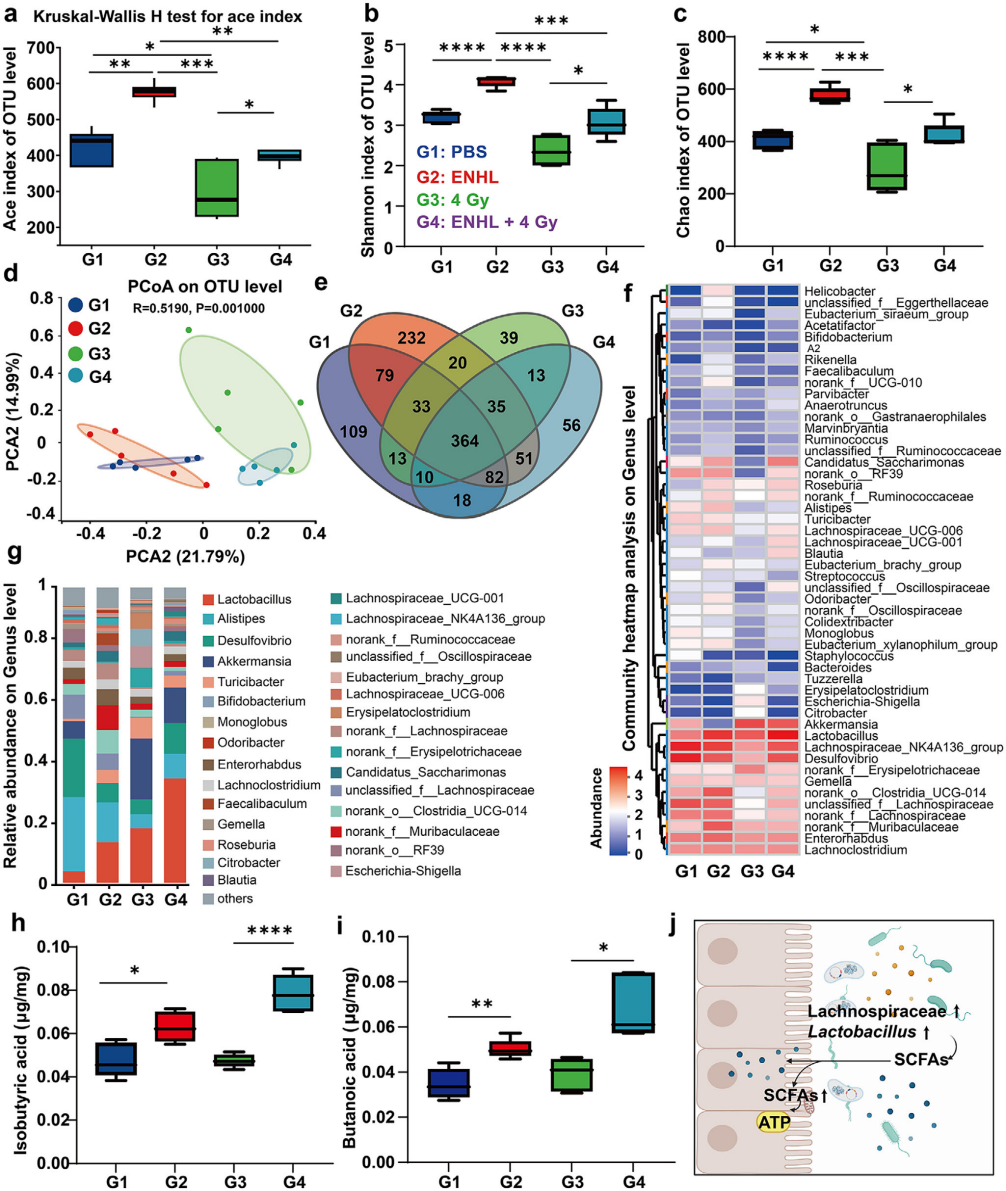
Regulation of intestinal microbiota by ENHL. (a) Ace index at the OTU level, evaluated using the Kruskal–Wallis H test across treatment groups (G1: PBS control; G2: ENHL; G3: 4 Gy; G4: ENHL + 4 Gy) (b, c) α‐Diversity assessed via Shannon index (b) and Chao index (c). (d) Principal component analysis (PCoA) of gut microbiota composition. (e) Venn diagram illustrating the overlap of identified strains in fecal samples across different treatment groups in orthotopic CRC mice. (f) Heatmap showing the relative abundance of gut microbiota at Genus level. (g) Taxonomic bar plot showing genus‐level microbiota distribution. (h,i) Short‐chain fatty acid (SCFA) metabolic analysis: isobutyric acid (h) and butyric acid (i) among different treatment groups. (j) Schematic diagram illustrating the regulatory effects of ENHL on intestinal microbiota. Created with BioRender. All data represent mean ± SD (*n* = 5). Statistical significance was determined by one‐way ANOVA with Tukey's multiple‐comparisons test (*p < 0.05, ** *p* < 0.01, *** *p* < 0.001, **** *p* < 0.0001).

Accumulating evidence indicates that tumorigenesis and progression induce significant alterations in gut microbiota composition and ecological structure, which subsequently modulate the immune profile of the TME and influence antitumor immune responses [50, 51]. In this study, we analyzed the fecal microbiota of mice from each experimental group by 16S rRNA gene sequencing, thus providing a comprehensive assessment of the regulatory effects of ENHL on gut microbial communities. Experimental mice were randomly assigned to four treatment groups: PBS gavage (G1), ENHL gavage (G2), 4 Gy (G3), and ENHL gavage + 4 Gy (G4). ENHL was administered by oral gavage administration, with tumor irradiation (4 Gy dose) performed after a 12‐hour interval. As shown in Figure 7a, the ENHL treatment in a rectal cancer mouse model significantly increased microbial richness, while the RT group (4 Gy radiation treatment) caused a substantial decrease in bacterial diversity. With respect to alpha (a) diversity metrics, both the Chao and Shannon indices indicated a higher microbial diversity in the ENHL‐treated group (Figure 7b,c). Beta (b) diversity analysis confirmed that ENHL intervention significantly altered the overall composition of the gut microbiota (Figure 7d,e). Next, compared to the control group, the β‐diversity in the RT treated mice underwent significant changes, with principal coordinate analysis (PCoA) revealing distinct inter‐group clustering differences (Figure 7d). Similarly, further analysis highlighted the shared and unique microbial taxa between the groups by using a Venn diagram (Figure 7e). Notably, the microbiota profiles of the ENHL + 4 Gy group had a marked divergence from the single‐RT treatment group (Figure 7f). At the family and genus levels, ENHL + 4 Gy treatment was associated with characteristic shifts, including increased abundance of beneficial bacteria such as Lachnospiraceae and *Lactobacillus* (Figure 7g and Figure S34). In addition, in the ENHL + 4 Gy group, we also observed a significant enrichment of beneficial bacteria, including *Lactobacillus reuteri* and *Lactobacillus johnsonii* at the species level (Figure S35).

Given the established role of short‐chain fatty acids (SCFAs) as both disease biomarkers and therapeutic targets in colorectal cancer [52], we investigated whether microbiota remodeling influenced SCFA production. Compared to other groups, we found that fecal levels of isobutyric acid and butanoic acid were markedly increased in the ENHL + 4 Gy group (Figure 7h,i), along with increased valeric acid and hexanoic acid (Figure S36). Overall, while RT alone substantially depleted SCFA levels, ENHL co‐administration not only prevented this decline but also restored SCFA production (Figure 7j). Collectively, these findings suggested that ENHL + 4 Gy combination therapy exerted antitumor effects through dual mechanisms: (1) reshaping gut microbiota composition to favor beneficial taxa, and (2) enhancing production of immunomodulatory SCFAs.

### 2.7 ENHL Combined with Radiotherapy Suppressed Intestinal Tumorigenesis in *APC* ^Min/+^ Mice

**FIGURE 8:**
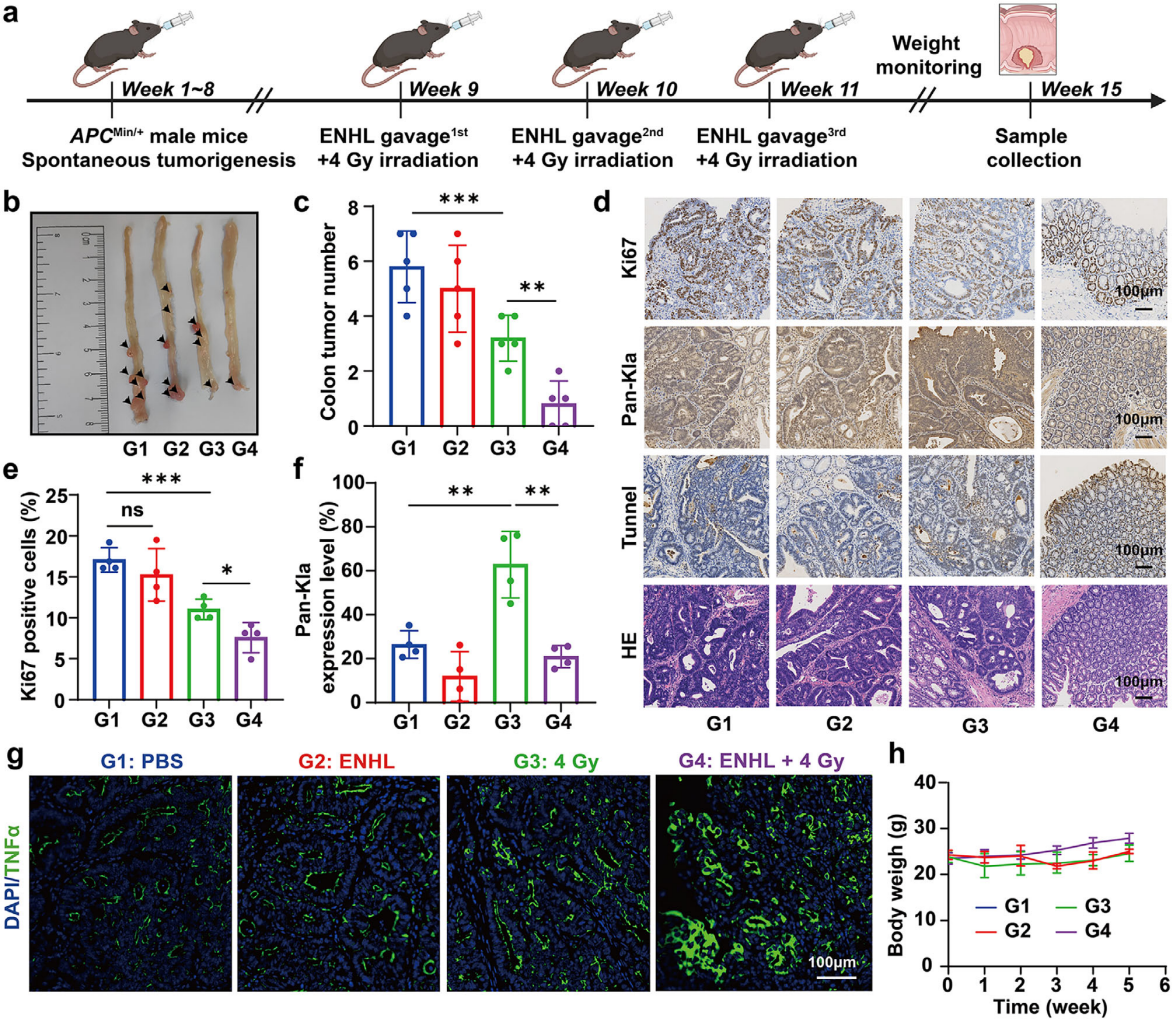
Anti‐tumor efficacy of the engineered bacteria in *APC* ^Min/+^ mice. (a) Schematic of the experimental design for treating *APC* ^Min/+^ mice. Created with BioRender. (b) Gross morphology of intestinal tumors across treatment groups. (c) Quantification of intestinal tumor burden in different treatment groups (*n* = 5). (d) Representative immunohistochemical staining of tumor sections for Ki67, Pan‐Kla, TUNEL, and H & E; scale bars = 100 µm. (e, f) Quantification of Ki67‐positive cells and Pan‐Kla staining intensity (*n* = 4). (g) Immunofluorescence staining of TNFα in intestinal tumors from *APC* ^Min/+^ mice under different treatments, scale bars = 100 µm. (h) Body weight changes in mice throughout the study (*n* = 5). All data represent mean ± SD. Statistical significance was determined by one‐way ANOVA with Tukey's multiple‐comparisons test (*p < 0.05, ** *p* < 0.01, *** *p* < 0.001, **** *p* < 0.0001, ns. not significant).

Building upon the previously demonstrated anti‐tumor efficacy of engineered bacteria combined with radiotherapy in an orthotopic rectal cancer model, we further evaluated their therapeutic potential in vivo using a spontaneous tumorigenesis mouse model with *APC* gene deficiency (Figure 8a). Experimental *APC* ^Min/+^ male mice were randomly assigned to four treatment groups: PBS (control, G1), engineered bacteria (ENHL, G2), radiotherapy (4 Gy, G3), and the combination treatment (ENHL + 4 Gy, G4). Each group received weekly cycles of oral gavage with the corresponding treatment, followed by 4 Gy of localized irradiation 24 h later (Figure 8a). Notably, the ENHL+ 4 Gy (G4) group exhibited a significant reduction in adenomas formation within the mid‐to‐distal colon compared to the other control groups (Figure 8b,c). Furthermore, we found that the combination treatment also significantly suppressed Ki67 expression and adenoma‐like transformation in *APC*‐deficient mice (Figure 8d,e). Importantly, the combined therapy mitigated radiation‐induced Pan‐Kla expression and resulted in the highest level of TUNEL positivity, signifying enhanced apoptosis (Figure 8d,f and Figure S37). To examine treatment‐induced changes in antitumor immunity, we performed immunofluorescence staining to quantify CD8⁺ T‐cell infiltration in intestinal tumors from *APC* ^Min/+^ mice receiving different therapeutic interventions. The results demonstrated that the combination treatment markedly increased the proportion of CD8⁺ T cells (Figure S38). Immunofluorescence staining also showed a pronounced upregulation of TNFα in the ENHL + 4 Gy group (Figure 8g and Figure S39). Moreover, mice receiving the combination therapy exhibited a steady and consistent trend of weight gain throughout the treatment course (Figure 8h). Taken together, these findings demonstrated that oral administration of engineered bacteria synergizes effectively with radiotherapy to elicit potent anti‐tumor responses in a spontaneous intestinal adenoma model with *APC* deficiency.

## 3 Discussion

RT has traditionally played a limited role in the management of colon cancer. Stereotactic body radiotherapy (SBRT), with its high‐precision targeting capability that minimizes damage to surrounding healthy tissues, has been increasingly utilized in selected cases of colon cancer (e.g., locally advanced disease or oligometastatic lesions) [53, 54, 55, 56]. Additionally, lactate plays a pivotal role in tumorigenesis and treatment resistance by influencing metabolic signaling pathways, modulating DNA damage repair proteins, and affecting lactylation levels in key proteins like PD‐L1 and p53 [32, 33, 57, 58, 59, 60]. Based on these findings, we hypothesized that radiosensitization could be achieved by reducing lactate levels in the tumor microenvironment. Currently, strategies aimed at modulating lactate in the TME primarily rely on inhibiting lactate production, such as through blockade of LDHA, or disrupting lactate transport, for example, by inhibiting monocarboxylate transporters (MCTs) [61, 62]. However, lactate‐targeting approaches based on small‐molecule inhibitors or drugs are often limited by short duration of action and poor tumor specificity, rendering them susceptible to metabolic reprogramming and compensatory bypass pathways; moreover, off‐target effects may lead to significant adverse reactions [63]. In addition, bacteria capable of metabolizing lactate or bacteria‐based hybrid systems have shown some potential in reducing lactate levels within the TME [64]. Nevertheless, lactate clearance in these systems depends heavily on the metabolic state and colonization efficiency of live bacteria and is typically achieved via intratumoral injection, with uncertain safety profiles and limited translational feasibility [65]. Engineered bacteria–nanomaterial hybrid systems are further constrained by complex designs, unclear translational pathways, and the frequent need to sacrifice long‐term bacterial functionality and colonization capacity, posing substantial challenges to long‐term biosafety [66, 67]. Importantly, most of these strategies are largely restricted to the extracellular lactate and are insufficient to effectively intervene in intracellular lactate or lactate‐driven protein lactylation processes.

Against this backdrop, probiotics that have already entered clinical trials, such as EcN, owing to their well‐established clinical safety and tumor‐targeting capabilities, have emerged as highly attractive therapeutic delivery vehicles [19, 27, 68]. Vesicle‐hyperproducing bacteria can generate large quantities of bacterial OMVs, and engineered OMVs carrying therapeutic cargos are capable of traversing the intestinal epithelial barrier to exert biological effects [27]. To selectively deplete lactate within the TME, we introduced LOx as a key enzyme for lactate degradation. Although various LOx‐based lactate‐depleting material systems have been reported, they are often hampered by suboptimal biocompatibility and limited clinical translational potential [14]. Accordingly, we developed an engineered “bacteria–vesicle” delivery system that integrates tumor‐targeted colonization, externally controllable release, inducible protein expression, and in situ OMV‐mediated intracellular lactate depletion. In this system, live bacteria continuously localize within the TME, where they not only sustain local LOx expression but also persistently generate LOx‐loaded OMVs. These LOx‐containing OMVs can deeply penetrate tumor tissues and enzymatically degrade lactate over a broad spatial range; importantly, they can also be internalized by tumor cells, thereby enabling dual regulation of both extracellular and intracellular lactate. Collectively, this strategy results in a marked enhancement of radiotherapeutic efficacy. Compared with bacteria that rely on endogenous lactate metabolism, vesicle‐hyperproducing engineered bacteria expressing LOx offer pronounced advantages in terms of externally controllable expression, expanded spatial reach (as OMVs, serving as nanoscale carriers, can access deep tumor regions and intracellular compartments that bacteria cannot readily reach, enabling synchronized extracellular and intracellular lactate clearance), immunomodulatory synergy, biosafety, and functional extensibility. This approach not only substantially improves the spatiotemporal controllability of lactate intervention but also establishes a direct mechanistic link between lactate depletion, suppression of DNA repair protein lactylation, and radiosensitization. In addition, oral administration of probiotics significantly increased gut microbiota diversity and short‐chain fatty acid levels, which may further help mitigate radiation‐induced adverse effects.

Despite these promising results, several limitations remain. First, although the preclinical model demonstrated effective tumor colonization and radiosensitization, and the engineered bacteria exhibited excellent long‐term safety in the host, its effect in humans still requires validation. Second, while arabinose‐induced gene expression systems perform well under controlled conditions, clinical translation may be hindered by interindividual variability in intestinal absorption and systemic distribution. In future research, we will focus on optimizing the system of intelligent responsiveness by developing gene expression systems regulated by endogenous signals from the tumor microenvironment (e.g., hypoxic conditions) and exogenous inducers, thereby enhancing the autonomy and precision of the treatment [69, 70]. We will also explore the engineering modification of EcN and OMVs, aiming to enhance vesicle stability, improve drug loading efficiency, and optimize their specific fusion ability with tumor cells by modulating their membrane composition and surface characteristics.

In summary, we developed an integrated biology platform that combined three core functions through precise gene circuit programming: specific tumor colonization, inducible protein expression system, and in situ bio‐delivery via bacterial outer membrane vesicles. This system simultaneously addresses multiple mechanisms of radioresistance by reprogramming tumor metabolism, modulating the immunosuppressive microenvironment, and enhancing gut microbial diversity while maintaining an excellent safety profile. The modular architecture of this microbial therapeutic platform integrates precise metabolic targeting with biomolecular delivery capabilities, offering a clinically transformed approach to overcome tumor radioresistance. The inherent adaptability of this platform makes it potentially applicable to diverse malignancies with distinct metabolic features, particularly pancreatic cancer and glioblastoma. Meanwhile, building on the clinical safety profile of EcN strains, this platform offers a novel therapeutic strategy for cancer therapy, and holds significant clinical translational potential.

## 4 Materials and Methods

### 4.1 Materials

Lactic Acid (LA) Content Assay Kit (Solarbio, BC2235), CheKine Micro Pyruvate Acid (PA) Assay Kit (Abbkine, KTB1121), FLAG‐Tag ELISA Kit (FineTest, EU2607), Annexin V‐APC Apoptosis Detection Kit (BioGems, 62700‐80‐Kit), TRITC Phalloidin (Solarbio, CA1610), Recombinant DYKDDDDK tag protein (Proteintech, Ag2329), and FastPure EndoFree Plasmid Maxi Kit (Vazyme, DC202‐01). Mycoplasma contamination was eliminated using MycAway Treatment (Yeasen, 40607ES03). Protein samples were prepared with 5X SDS‐PAGE Loading Buffer (Affinity Biosciences, AIWB‐0025), and nuclei were counterstained with Anti‐Fade Mounting Medium containing DAPI (Beyotime, P0131‐5 mL). Cell viability was assessed using the Cell Counting Kit‐8 (Beyotime, C0037). Primary antibodies included anti‐Syndecan‐1 antibody (Abcam, ab128936), anti‐Dnak antibody (Abcam, ab69617), Anti‐L‐Lactyl Lysine Rabbit mAb (PTM Bio, 9H1L6), Ki‐67 Polyclonal antibody (Proteintech, 28074‐1‐AP), HIF1α Rabbit pAb (ABclonal, A11945), LDHA Rabbit pAb (ABclonal, A1146), β‐Actin Rabbit mAb (ABclonal, AC050), DDDDK‐Tag Rabbit mAb (ABclonal, AE092), Myc‐Tag Rabbit mAb (ABclonal, AE070), GAPDH Rabbit mAb (ABclonal, A19056), MRE11 (Boster, BM5057), and Anti‐gamma H2A.X (Abcam, ab81299). Secondary antibodies included Cy3‐conjugated Goat anti‐Rabbit IgG (ABclonal, AS007) and Alexa Fluor 488 (Thermo Fisher, A‐11008). Key reagents were obtained from MedChemExpress, Lactate Oxidase (HY‐134757), L‐Lactic Acid (HY‐Y0479), and Heparin (HY‐17567). For flow cytometry, the following anti‐mouse antibodies were used: CD3‐FITC (Biolegend, 100202), CD4‐APC (Biolegend, B201509), CD8‐PE (Biolegend, 100734), CD11c‐FITC (Elabscience, N418), CD86‐APC (Biolegend, 105008), CD80‐PE (Biolegend, 104708), CD11b‐PerCP (Elabscience, E‐AB‐F1081F), and F4/80‐FITC (Elabscience, E‐AB‐F0995C), CD8 alpha (RPA‐T8) Mouse Monoclonal Antibody FITC Conjugate (Cell signaling, 55397).

### 4.2 Cell Lines

CT26, HCT116, and CT26‐Luc cell lines were obtained from the Cell Bank of the Chinese Academy of Sciences (Shanghai, China). All cell lines were maintained in RPMI‐1640 Medium (Procell system, PM150110) or high‐glucose DMEM (Procell system, PM150210) supplemented with 10% Fetal Bovine Serum (Vazyme, F101‐03) and 1% penicillin‐streptomycin (NCM Biotech, C100C5) at 37°C in a 5% CO_2_ humidified atmosphere.

### 4.3 Animal and Tumor Models

Female C57BL/6 and BALB/c mice (6–8 weeks old), and male B6/JGpt‐*APC* ^em1Cin (Min)^/Gpt mice (8 weeks old) were obtained from GemPharmatech Co., Ltd. All procedures were approved by the Soochow University Animal Ethics Committee (Ethics Number: 202408A0372). Mice were maintained under specific pathogen‐free (SPF) conditions at 20°C–22°C with 30%–70% humidity and a 12 h light/dark cycle. For orthotopic tumor models of low rectal cancer, CT26‐Luc cells (2 × 10^6^) scattered in 50 µL PBS were inoculated into the rectal mucosa of BALB/c mice, and tumor growth was subsequently monitored using IVIS Imaging System (IVIS Lumina Series III, USA).

### 4.4 Patients and Tumor Samples

Tumor specimens were collected from patients with pathologically and clinically confirmed colorectal cancer at the First Affiliated Hospital of Soochow University. Prior to sample collection, written informed consent was obtained from all participants. The Ethics Committee of the First Affiliated Hospital of Soochow University reviewed and approved the study protocol (Approval Notice No. 2025‐702). Relevant clinical characteristics, including age, sex, and tumor size, were extracted from the hospital's clinicopathological database and are summarized in Table S1. Treatment response was assessed using the Mandard Tumor Regression Grade (TRG) system. Patients demonstrating complete pathological response (TRG0) or marked regression (TRG1) were classified as the sensitive group, whereas those with partial regression (TRG2) or minimal/no regression (TRG3) were categorized as the resistant group.

### 4.5 Plasmid Construction and Bacterial Culture

The probiotic *Escherichia coli* Nissle 1917 (EcN) strain and its derivative mutant with the *nlpI* gene deletion (EcNΔ*nlpI*; constructed by Bosai Biotech) were used in this study. Three engineered strains were generated: (1) EcNΔ*nlpI* ^EG^ (ENGP), created by transforming EcNΔ*nlpI* with the pBAD‐His‐Myc‐EGFP (EG) plasmid; (2) EcNΔ*nlpI* ^IHCG^ (ENHP), constructed using the pBAD‐His‐Myc‐INP‐HlpA‐RBS‐ClyA‐EGFP‐3×Flag (IHCG) plasmid; and (3) EcNΔ*nlpI* ^IHCL^ (ENHL), generated by transforming EcNΔ*nlpI* with the pBAD‐His‐Myc‐INP‐HlpA‐RBS‐ClyA‐EGFP‐3×Flag (IHCL) plasmid. All plasmids were synthesized by Ansenda (Suzhou, China) and their sequences information is in Table S2. Bacterial cultures were grown in LB medium at 37°C with shaking (220 rpm), supplemented with ampicillin (50 µg/mL) as needed.

### 4.6 OMV Preparation and Characterization

Bacterial cultures were then centrifuged (4°C, 4000 rpm, 20 min), and the supernatant was sequentially filtered through a 0.22 µm vacuum filter (Wuxi Nice Biotechnology, 343011) and concentrated using a 100 kDa ultrafiltration membrane (Millipore, uFC905008). Additional purification was performed using a 0.22 µm disposable syringe filter. The resulting filtration was ultracentrifuged (150 000 g × 3 h, 4°C), and the pelleted OMVs were resuspended in 1 mL PBS (from an initial 1000 mL culture volume) and stored at −20°C. OMV protein concentration was quantified using a BCA protein assay kit (Nanjing Novozan Biotechnology, E112‐01). Morphological characterization was performed by cryo‐electron microscopy (Aifang Biotechnology), while particle size distribution and zeta potential were analyzed using a Zetasizer Nano ZS90 system (Malvern, UK).

### 4.7 Western Blot Analysis

The isolated OMVs were lysed in 4:1 RIPA buffer supplemented with 5× loading buffer, followed by denaturation at 90°C for 15 min. Protein samples were separated by 10% SDS‐PAGE and subsequently transferred to NC membranes. After blocking with 5% skim milk for 1 h at room temperature, membranes were incubated overnight at 4°C with primary antibodies. Following TBST washes, membranes were probed with HRP‐conjugated goat anti‐rabbit/Mouse secondary antibody (ABclonal, AS031) for 1 h at room temperature. Protein bands were visualized using enhanced chemiluminescence reagents and imaged with the Tanon 5200 Fully Automated Chemiluminescence Imaging System (Tanon Science & Technology Co., Ltd.).

### 4.8 In Vitro Endocytosis Experiments

To assess the endocytic capacity of CT26 cells for OMVs‐P, cells were plated in confocal dishes at a density of 1 × 10⁴ cells per well and exposed to 100 µg EGFP‐labeled OMVs‐P for 24 h. After incubation, non‐internalized bacteria were removed through three PBS washes, and cellular uptake was subsequently analyzed by fluorescence imaging using an Olympus FV1200 confocal microscope.

### 4.9 Cell Survival Assay

To perform cell viability assays, experimental cells (1000 cells per well) are seeded in 4 or 5 replicates in 96‐well plates and incubated overnight at 37°C and 5% CO_2_. The indicated vesicles were added 6 h prior to 6 Gy X‐ray irradiation, and the cells were cultured in their respective media for 24 h. Cell viability was assessed using CCK8 (Dojindo, CK04) and absorbance was measured at 570 nm or 450 nm using the BioTek plate reader, respectively.

### 4.10 Optimization of Arabinose Induction Conditions

In vitro, different concentrations of Ara (0, 0.5, 1, 2, and 4 g/L) were used to induce under the same amount of bacterial solution, and the fluorescence intensity of bacterial solution was detected by microplate reader for 8 h after induction. In vivo, a dose of 1 × 10⁹ bacteria was administered to each mouse by gavage, and then after induction with different concentrations of arabinose for 12 h, all the feces of the mice were collected, and then they were diluted with 1 mL PBS, and then placed in a microplate reader to detect the fluorescence intensity.

### 4.11 Intestinal Distribution of Engineered Bacteria

To evaluate the intestinal distribution and colonization dynamics of engineered bacteria, healthy BALB/c mice (*n* = 3) were randomly assigned to five groups and administered 100 µL of bacterial suspension (1 × 10⁹ CFU in 20 g/L arabinose solution) via oral gavage. The mice were euthanized at predetermined time points (0, 2, 5, 12, and 24 h post‐administration), followed by complete extraction of gastrointestinal tracts. Bacterial retention in the colon region was quantitatively assessed using an IVIS imaging system (IVIS Lumina Series III, USA) in vivo imaging system.

### 4.12 Enzyme Kinetic Assays

LOx activity was determined by quantifying pyruvate generation during lactate oxidation. Briefly, the reaction was initiated by adding an LOx sample (0.5 mg/mL) to a PBS solution containing Lactate substrate (0.05 mg/mL). The resulting pyruvate production was then measured using a commercial pyruvate assay kit (Solarbio, BC2200), with kinetic measurements obtained by continuously monitoring absorbance at 520 nm using a microplate reader.

### 4.13 Lactate Concentration Determination

The lactate concentration was determined by the lactate detection kit (Solaibao, BC2230). All tissue samples were added to the extract at a ratio of 1:5‐10 (mass/volume), and the supernatant was obtained by centrifugation at 12 000 g at 4°C for 10 min after homogenization in an ice bath; cell supernatant samples were added to the extract at a ratio of 100 µL/mL. During the test, the sample control tube and the sample determination tube are set up, and the standard quality control is set in the same way. After 30 min of incubation at 37°C, the absorbance was measured at 570 nm using a microplate reader, and the ΔA value was calculated.

### 4.14 Bacterial Colonization and LOx Expression

The tumor‐targeted colonization efficiency and LOx expression level of oral engineered EcN were systematically evaluated, and the tumor tissue was obtained 24 h after administration using a gradient dose administration regimen, and the bacterial colonization amount (CFU/g tissue) was quantified by plate coating method (Ampicillin LB medium). At the same time, the Flag‐tagged ELISA kit (FineTest, EU2607) was used to detect the expression level of LOx‐Flag fusion protein in tumor homogenate.

### 4.15 In Vivo Imaging of CT26‐Bearing Mice

To monitor tumor development, we utilized luciferase‐expressing CT26 cells (CT26‐Luc). At specified observation time points, mice were intraperitoneally injected with D‐luciferin sodium salt (5 mg/mL in PBS) (YEASEN, 40901ES01). Bioluminescence imaging was performed 10 min post‐injection using the IVIS Spectrum imaging system.

### 4.16 In Vivo Antitumor Therapy

The engineered bacteria (1 × 10^9^ CFU) were administered by gavage, followed by isoflurane anesthetized and mechanically fixed in a special fixture for X‐ray irradiation (radiation dose rate: 1.375 Gy/min, RS‐2000 Pro irradiator). In this process, the researchers used precise positioning to ensure that only the tumor site was exposed to the radiation field, while the rest of the mice's body were effectively protected. After one day of treatment, the mice were euthanized, and tumor tissues were harvested for subsequent analysis. Immunofluorescence staining and flow cytometry were employed to analyze the infiltration and abundance of immune cells within distant tumor tissues. Concurrently, tumor specimens underwent H&E staining and relevant immunohistochemistry assay to evaluate histopathological alterations and apoptotic activity. The remaining cohort of mice was subjected to longitudinal monitoring to assess survival outcomes.

### 4.17 Immunofluorescence (IF) Staining

Fresh tumor tissues were immediately embedded in OCT medium, flash‐frozen in liquid nitrogen, and stored at −80°C. For sectioning, frozen blocks were cryosectioned at 5–10 µm thickness, mounted onto pre‐chilled slides, and air‐dried for 5–10 min. Sections were either fixed (4% PFA, 10 min) or stored at −80°C for later use. For immunofluorescence, sections were washed with PBST, blocked with 5% normal goat serum, and incubated with primary antibodies overnight at 4°C, followed by fluorescent secondary antibodies. After mounting with aqueous medium, images were acquired using fluorescence microscopy.

### 4.18 16S rRNA Gene Amplicon Sequencing of Fecal Microbiota

Five days after the completion of all intervention treatments in each group, the cecal contents were aseptically collected, immediately aliquoted in pre‐cooled sterile cryopreservation tubes, and transferred to an ultra‐low temperature freezer at −80°C for storage after liquid nitrogen quick‐freezing. All samples were sent to the testing platform of Meiji Biotechnology Co., Ltd. (Shanghai, China) through professional cold chain transportation (dry ice and cold storage at a temperature of ≤−70°C) for the following purposes: 1) sequencing of the V3‐V4 region of the 16S rRNA gene on the Illumina NovaSeq platform to analyze the composition of intestinal microbiota; 2) Gas chromatography‐mass spectrometry (GC‐MS) was used to quantitatively detect the content of short‐chain fatty acids (including acetic acid, propionic acid, butyric acid, etc.).

### 4.19 Biosafety Evaluation

BALB/c mice were acclimatized for 7 days and randomly divided into two groups (healthy group and ENHL group. Mice are orally administered ENHL (1 × 10^9^CFU/two days) for 4 weeks. After that, blood samples are collected for routine blood tests and biochemical analysis.

### 4.20 Statistical Analysis

All values reported in this study are presented as mean ± standard deviation (SD). Intergroup comparisons were performed using Student's *t*‐tests, and multi‐group analyses were conducted using one‐way ANOVA with Tukey's multiple‐comparisons test. Statistical analyses were performed using GraphPad Prism (version 10.0) and Microsoft Excel (2020). The threshold for statistical significance was established at *p* ≤ 0.05, with specific p‐values and corresponding statistical tests detailed in respective figure captions.

## Author Contributions

F.P. and Z.L. conceptualized the study and designed the methodology. Z.Z. and Z.S. synthesized and characterized the materials. H.Y. and S.L. performed in vitro experiments. C.Z. conducted in vivo investigations. M.H. and Y.W. analyzed bioinformatics data. L.G., L.H., and K.Y. supervised the research, acquired funding, and provided project administration, served as the corresponding author.

## Conflicts of Interest

The authors declare no conflicts of interest.

## Supporting information




**Supporting File**: advs73681‐sup‐0001‐SuppMat.docx.

## Data Availability

The datasets generated and analyzed during this study are available from the corresponding author upon reasonable request.
