# Supplementary material for: Engineered Bacteria‐Vesicle Delivered Lactate Reprogramming Boosts Tumor Radiosensitivity
Source: Adv Sci (Weinh). 2026 Jan 4;13(16):e24303. doi: 10.1002/advs.202524303 (PMC13042793; doi:10.1002/advs.202524303)
Supplement: Supplementary file 1 — Supporting File: advs73681‐sup‐0001‐SuppMat.docx. [file ADVS-13-e24303-s001.docx]

**Supporting information**

**Engineered Bacteria-Vesicle Delivered Lactate Reprogramming**

**Boosts Tumor Radiosensitivity**

*Fei Peng^#^, Zhe Lei^#^,* *Zhehao Zhang,* *Zhiyue Su,* *Chonghai Zhang,* *Huan Yang, Shu Liu,* *Mengyuan Hu, Yuhong Wang,* *Lingchuan Guo*, Lin Hu*, Kai Yang**

Prof. K. Yang, Prof. L. Hu, Prof. L. Guo, Prof. Z. Lei, Dr. Z. Su, Miss. F. Peng, Y. Wang, M. Hu, Mr. H. Yang, Z. Zhang, C. Zhang, S. Liu.

Department of Pathology, the First Affiliated Hospital, State Key Laboratory of Radiation Medicine and Protection, School of Radiation Medicine and Protection & School for Radiological and Interdisciplinary Sciences (RAD-X), Collaborative Innovation Center of Radiation Medicine of Jiangsu Higher Education Institutions, Cancer Institute, Suzhou Medical College, Soochow University, Suzhou, Jiangsu 215123, China.

Corresponding authors: szglc@hotmail.com, hulin@suda.edu.cn, [kyang@suda.edu.cn](mailto:kyang@suda.edu.cn)


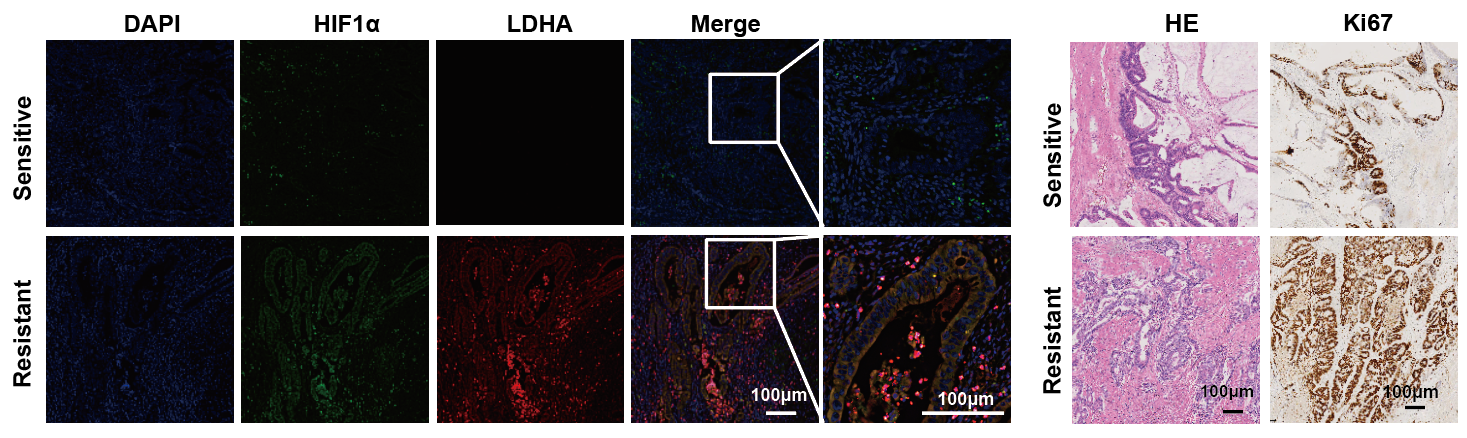


**Figure S1**. Immunofluorescence analysis of LDHA and HIF-1α expression, and immunohistochemical staining of H&E and Ki67 in colorectal cancer tissues from 6 cases sensitive to neoadjuvant radio-chemotherapy and 12 cases resistant to neoadjuvant radio-chemotherapy. scale bar, 100 μm.


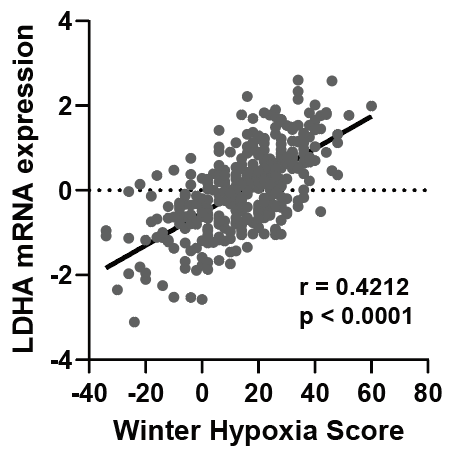


**Figure S2**. The correlation analysis of LDHA mRNA expression with hypoxia signature scores in colorectal cancer (r = 0.4212, *P* < 0.0001). Analysis was performed using the cBioPortal for Cancer Genomics (https://www.cbioport-al.org/). Gene expression profiles and clinical data were derived from the TCGA PanCancer Atlas (Colorectal Adenocarcinoma dataset).


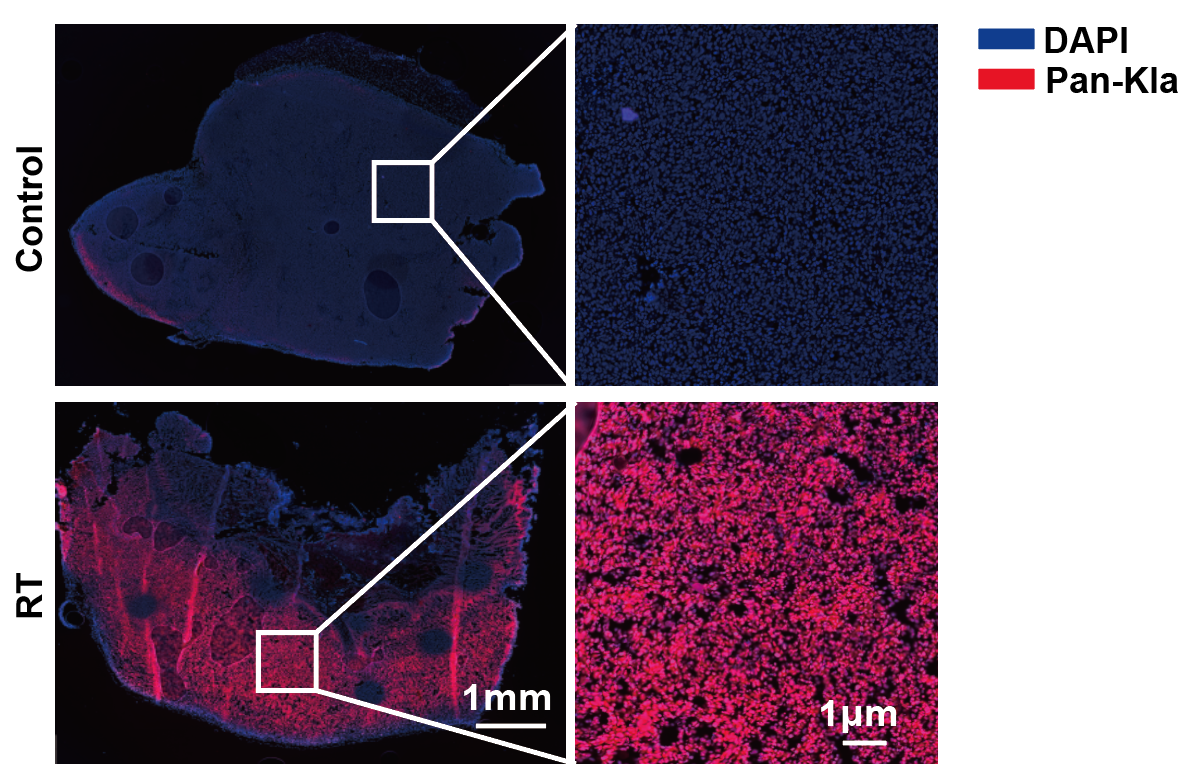


**Figure S3**. Representative immunofluorescence images of Pan-Kla staining in CT26 subcutaneous tumors from mouse models. Pan-Kla (red), and DAPI-labeled nuclei (blue); scale bar, 100 μm (RT; 6 Gy/fraction × 3 fractions, administered every 2 days).


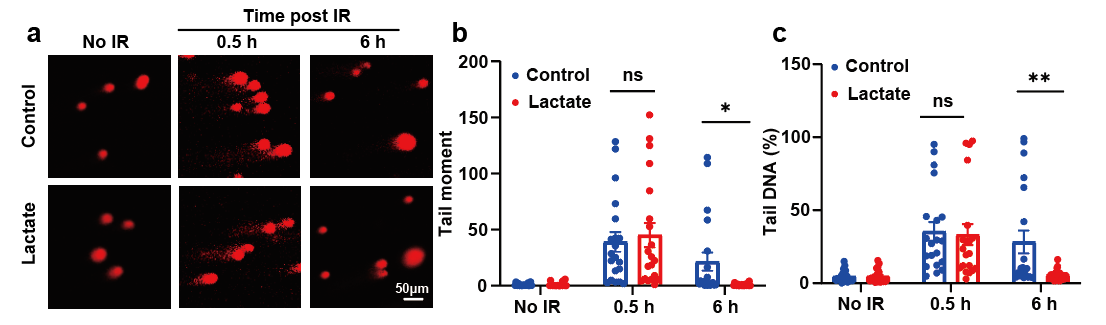


**Figure S4**. (**a**) Representative Comet assay images of CT26 cells pretreated with/without 20 mM lactate for 24 h followed by 10 Gy irradiation, harvested at indicated timepoints; scale bar: 50 μm. (**b, c**) Quantification of tail moment and tail DNA percentage (n = 20 randomly selected cells per group). All data represent Mean ± SD. Statistical significance was determined by Student’s t-test (**P* < 0.05, ***P* < 0.01, ****P* < 0.001, *****P* < 0.0001, ns. not significant).


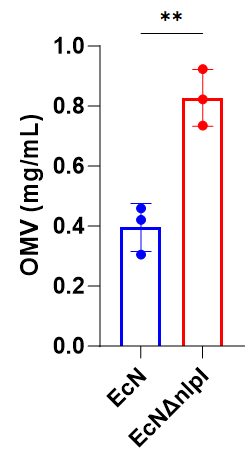


**Figure S5**. OMVs production in EcN vs. EcNΔ*nlpI*. BCA assay quantification revealed significantly higher OMVs yield in EcNΔ*nlpI* (2.09 ± 0.49-fold) versus wild-type EcN. Mean ± SD (n = 3). Statistical significance was determined by unpaired two-tailed Student’s t-test (**P* < 0.05, ***P* < 0.01).


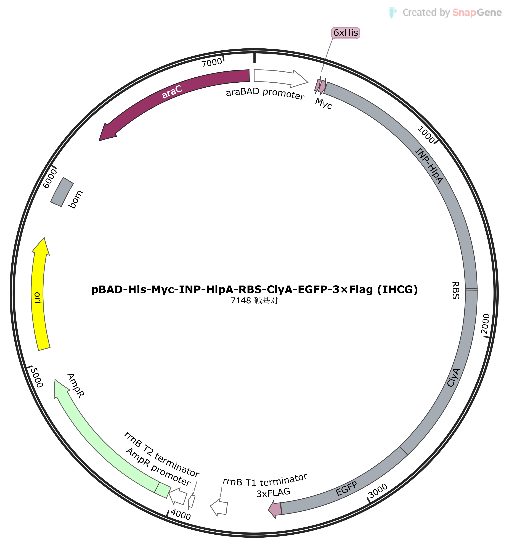

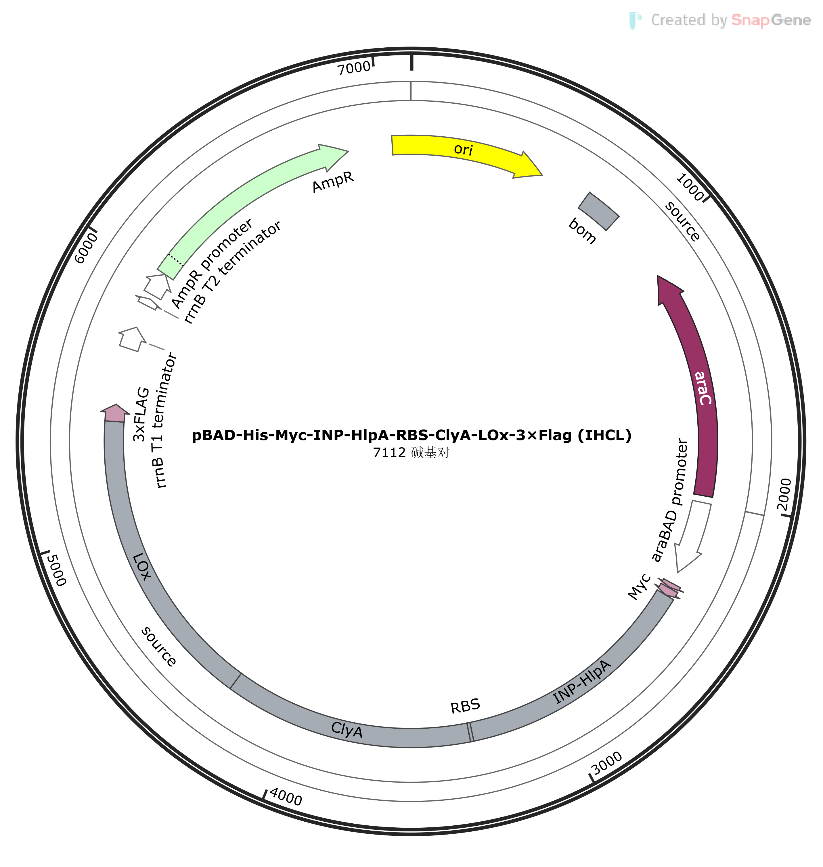


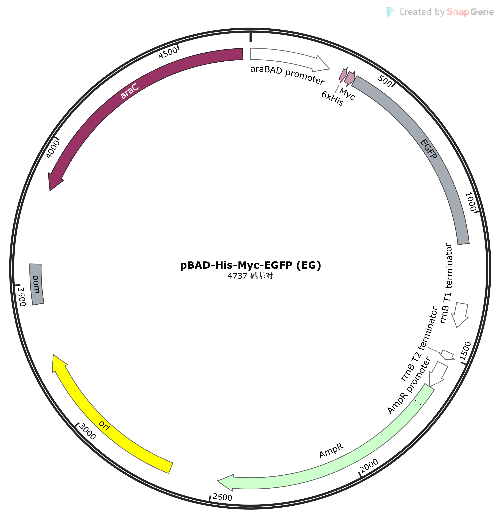


**Figure S6.** Plasmid vector map, including the plasmid IHCG (pBAD-His-Myc-INP-HlpA-RBS-ClyA-EGFP-3×Flag), the plasmid EG (pBAD-His-Myc-EGFP) and the plasmid IHCL (pBAD-His-Myc-INP-HlpA-RBS-ClyA-LOx-3×Flag).


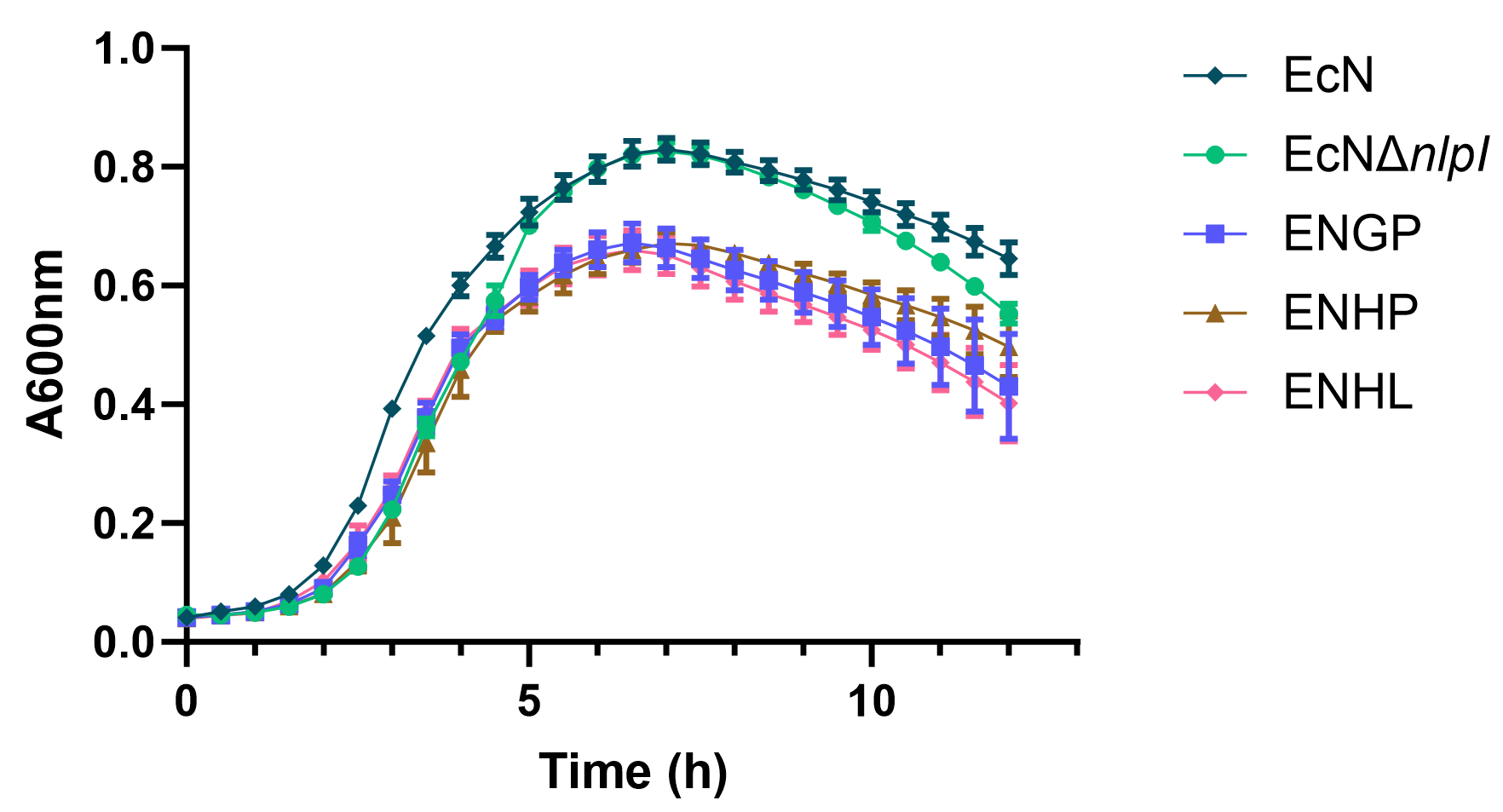


**Figure S7**. Growth curves of EcN and EcNΔ*nlpI* in LB medium, while ENGP, ENHP, and ENHL in LB medium with ampicillin (pH 6.8). The optical density of bacteria was monitored over time using a microplate reader at 600 nm. All strains were cultured in LB broth (pH adjusted to 6.8) at 37 °C with measuring every 30 minutes.


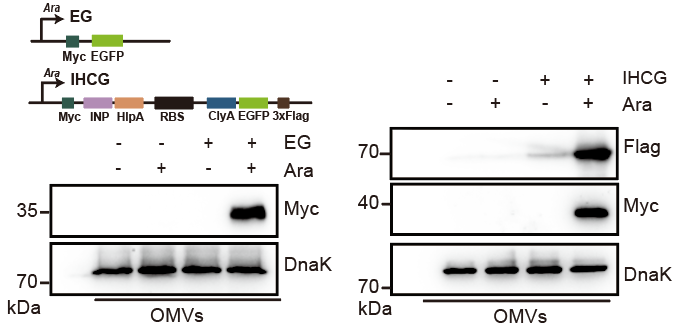


**Figure S8**. Western blot analysis was used to assess the expression of EG and IHCG in the engineered EcNΔ*nlpI* strains secreting OMVs, with arabinose (Ara) serving as the expression inducer.

**
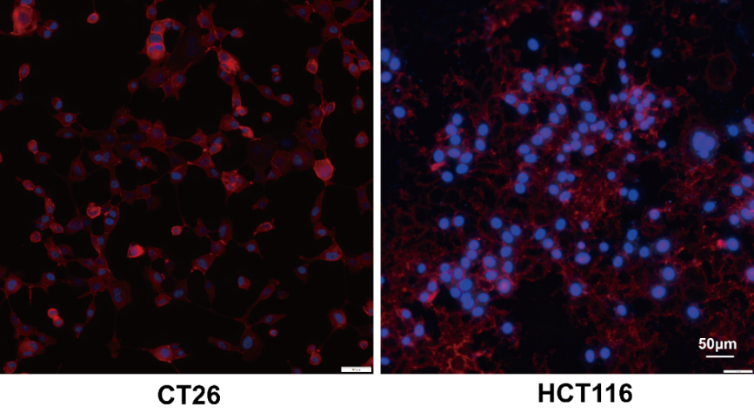
**

**Figure S9.** Heparin sulfate proteoglycan (HSPG) expression on the plasma membrane of the indicated cell lines, as observed by Olympus SLIDEVIEW VS200. HSPG was stained with anti-syndecan-1 antibody (red), while cell nuclei were stained with DAPI (blue). Scale bar, 50 μm.


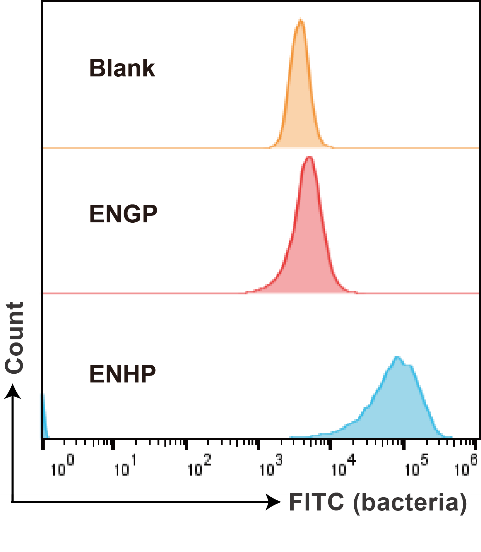


**Figure S10.** The tumor-targeting ability of ENHP. Engineered bacteria were incubated with the CT26 cells for 2 h at 4 °C, and the bacterial fluorescence intensities of the cells were examined using flow cytometry.


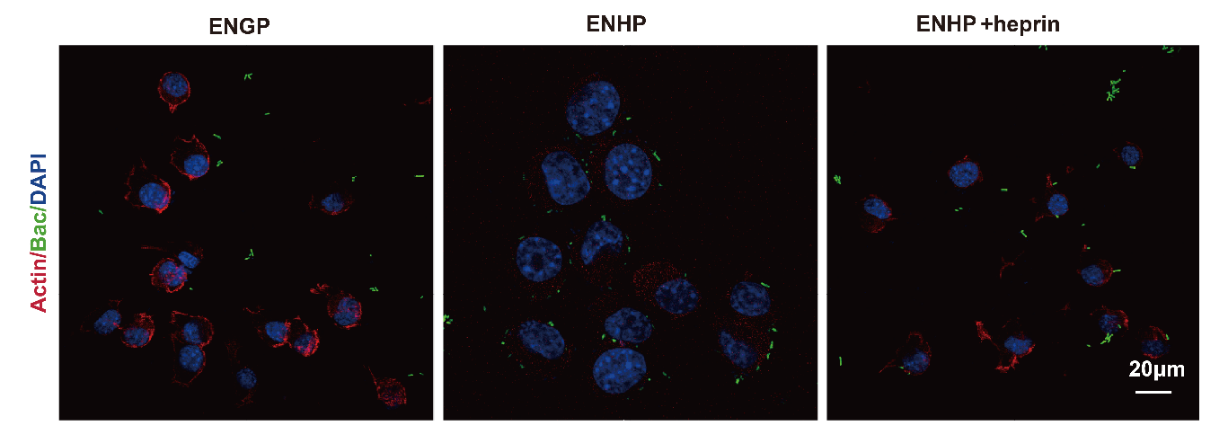


**Figure S11.** Representative fluorescence images demonstrating bacterial binding to CT26 cells. Bacteria (green; EGFP labeled), actin filaments (red; phalloidin), and nuclei (blue; DAPI); scale bar: 20 μm.


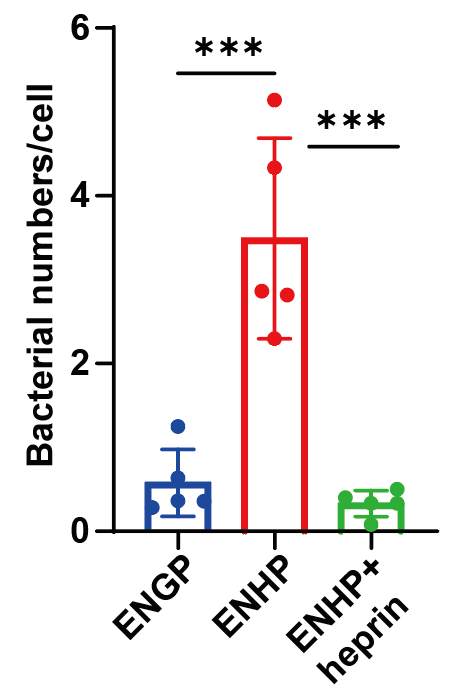


**Figure S12.** Quantification of the binding of bacteria onto HCT116 cells. Mean ± SD (n = 5). Statistical significance was determined by one-way ANOVA with Tukey’s multiple comparisons (**P* < 0.05, ***P* < 0.01, ****P* < 0.001).


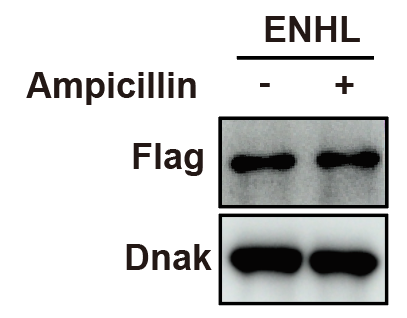


**Figure S13.** Representative Western blot results of Flag-tag in ENHL cultured with or without ampicillin for 24 h, showing no influence of ampicillin on the plasmid stability.


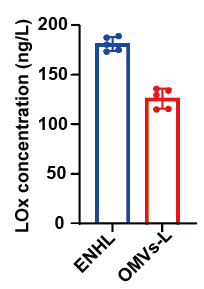


**Figure S14.** LOx concentration of ENHL and its OMVs as measured by LOx-ELISA (n = 5).


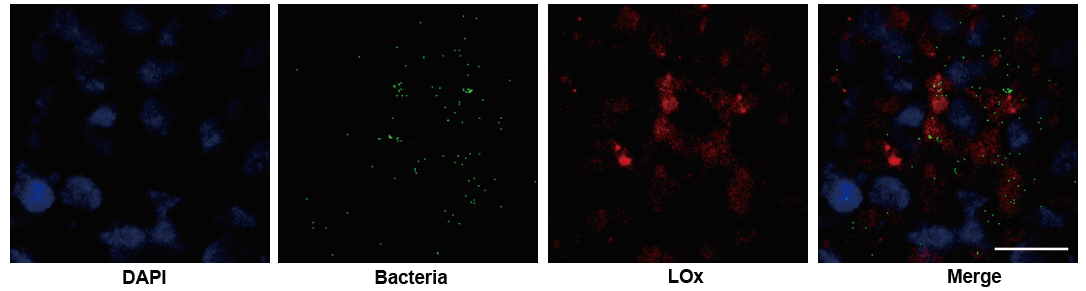


**Figure S15.** Representative images of tumor sections depicting bacterial localization and produced proteins; scale bar, 100 μm.


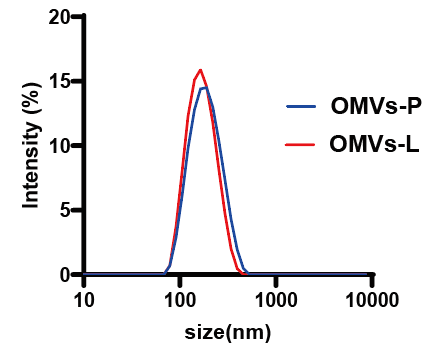


**Figure S16**. Hydrodynamic size of different OMVs detected by DLS.


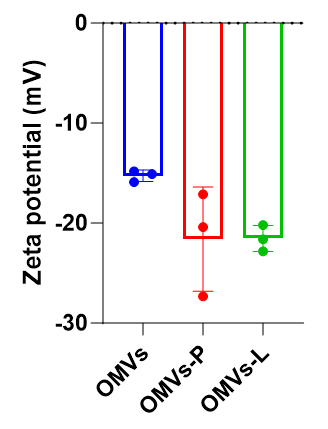


**Figure S17**. Zeta potential of different OMVs (n = 3).


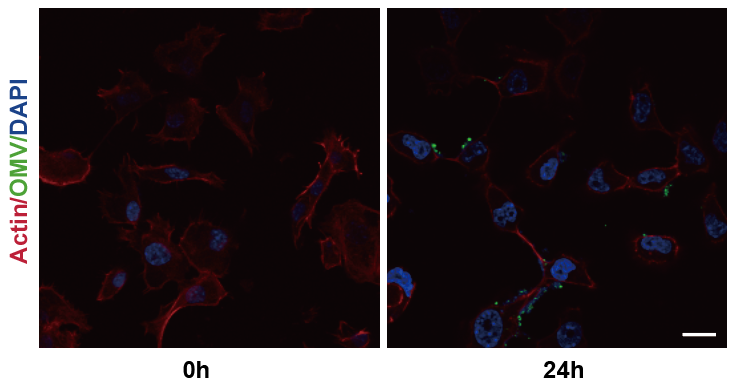


**Figure S18**. Representative confocal microscopy images showing the internalization of OMVs-P (green) by CT26 cells at 24 h post incubation. Actin (red) and nuclei (blue) were stained with phalloidin and DAPI, respectively; scale bar = 20 μm.


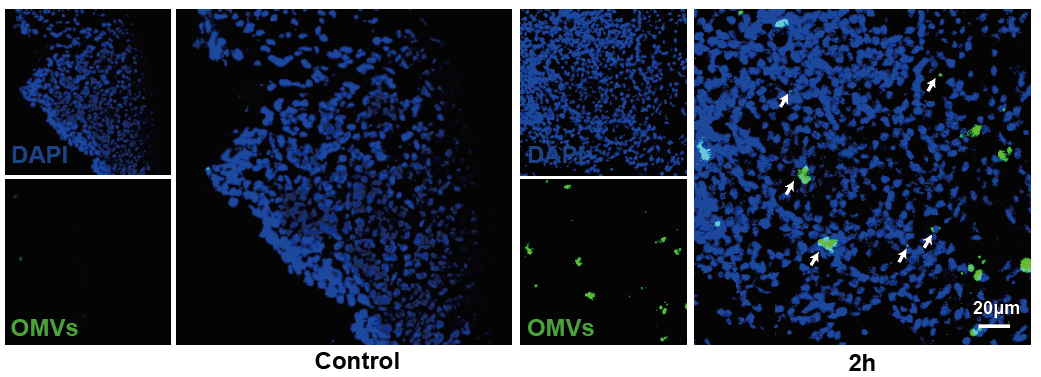


**Figure S19**. Intestinal epithelial permeability assay: GFP-labeled OMVs-P (green) were injected into ligated colonic segments and analyzed after 2 h. Nuclei were counterstained with DAPI (blue); scale bar = 20 μm.


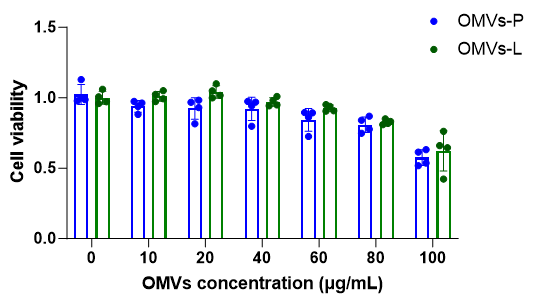


**Figure S20.** Cytotoxicity assessment of bacterial extracellular vesicles in CT26 cells. CT26 cell viability following 24-hour treatment with ENHP-derived OMVs (OMVs-P) or ENHL-derived OMVs (OMVs-L) was quantified using CCK-8 assay. Mean ± SD (n = 4).


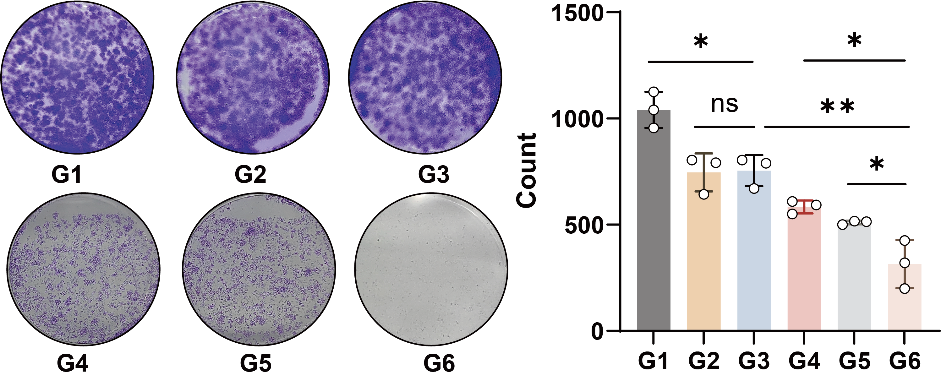


**Figure S21**. Representative cloning evaluation images of CT26 cells treated by different formulations (G1: PBS control; G2: OMVs-P; G3: OMVs-L; G4: 6 Gy; G5: OMVs-P + 6 Gy; G6: OMVs-L + 6 Gy; n = 3).


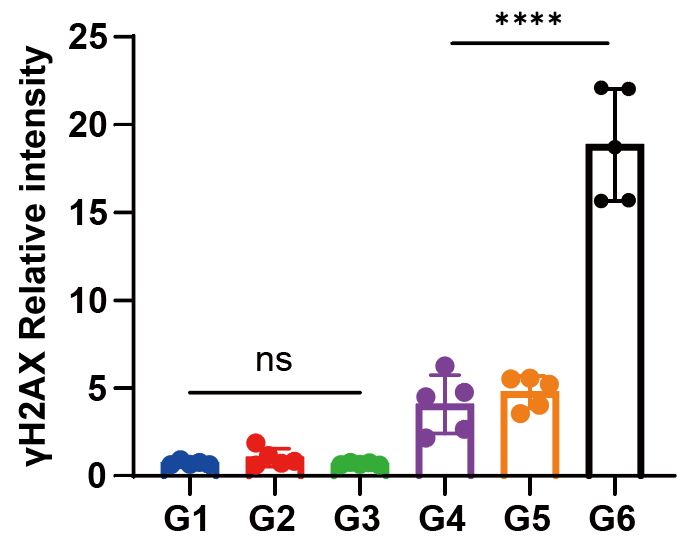


**Figure S22**. Quantification graphs of γH2AX images of CT26 cells treated by different formulations (G1: PBS control; G2: OMVs-P; G3: OMVs-L; G4: 6 Gy; G5: OMVs-P + 6 Gy; G6: OMVs-L + 6 Gy; Mean ± SD, n = 5). Statistical significance was determined by one-way ANOVA with Tukey’s multiple comparisons (**P* < 0.05, ***P* < 0.01, ****P* < 0.001, *****P* < 0.0001, ns. not significant).


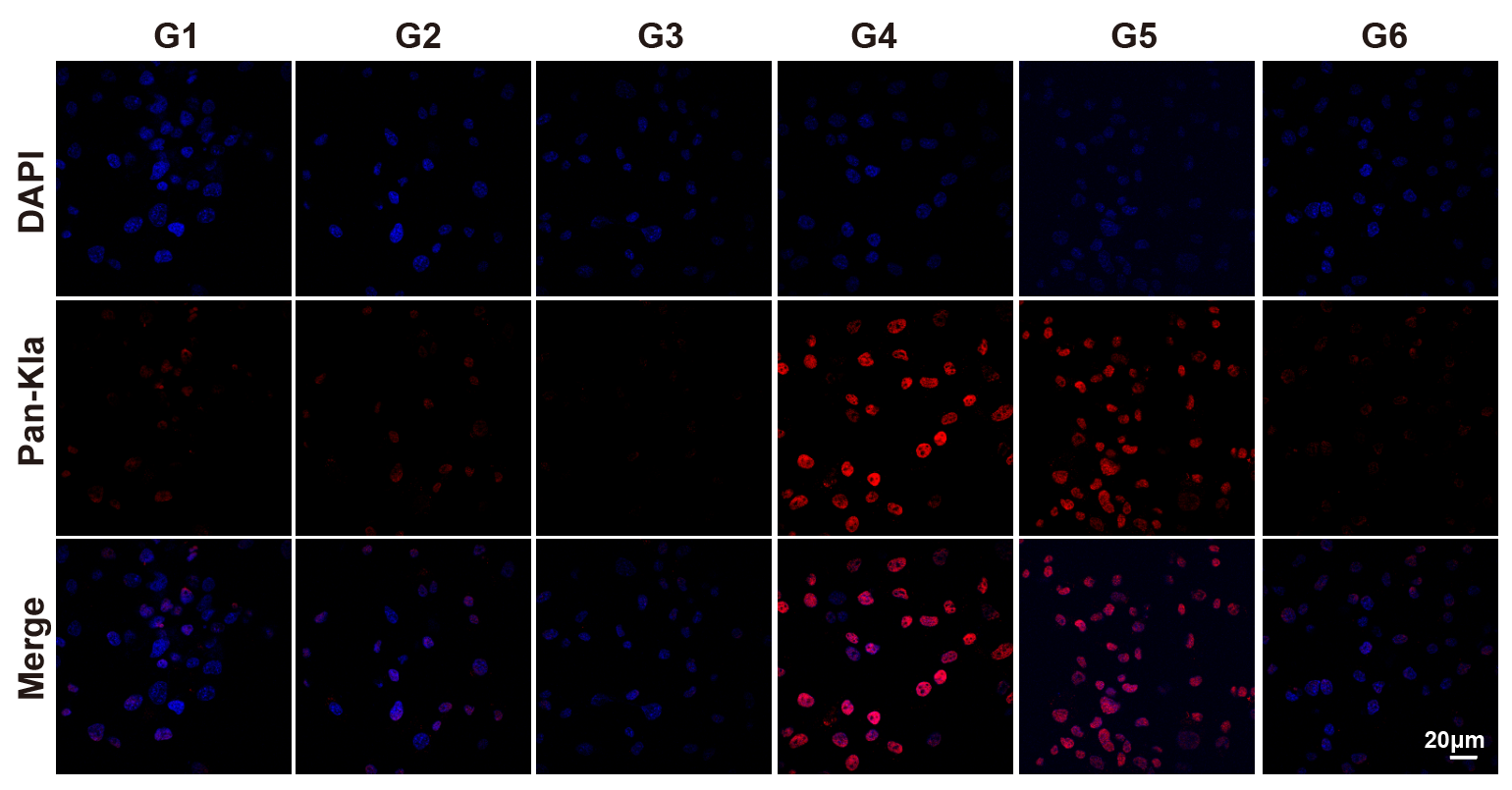


**Figure S23**. Immunofluorescence images of Pan-Kla staining after different treatments. DAPI (blue). Scale bar, 20 μm (G1: PBS control; G2: OMVs-P; G3: OMVs-L; G4: 6Gy; G5: OMVs-P + 6 Gy; G6: OMVs-L + 6 Gy; n = 5).


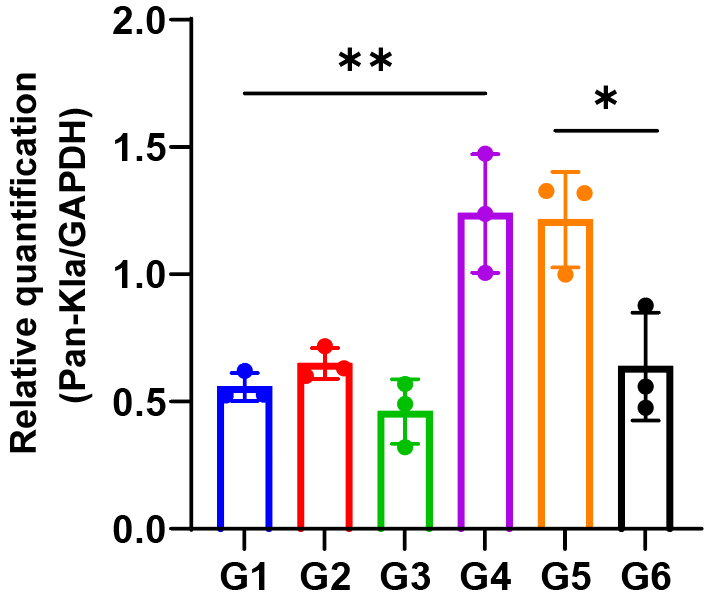


**Figure S24**. Relative quantification of Pan-Kla/GAPDH in different treatment groups (G1: PBS control; G2: OMVs-P; G3: OMVs-L; G4: 6 Gy; G5: OMVs-P + 6 Gy; G6: OMVs-L + 6 Gy; Mean ± SD, n = 3). Statistical significance was determined by one-way ANOVA with Tukey’s multiple comparisons (**P* < 0.05, ***P* < 0.01).


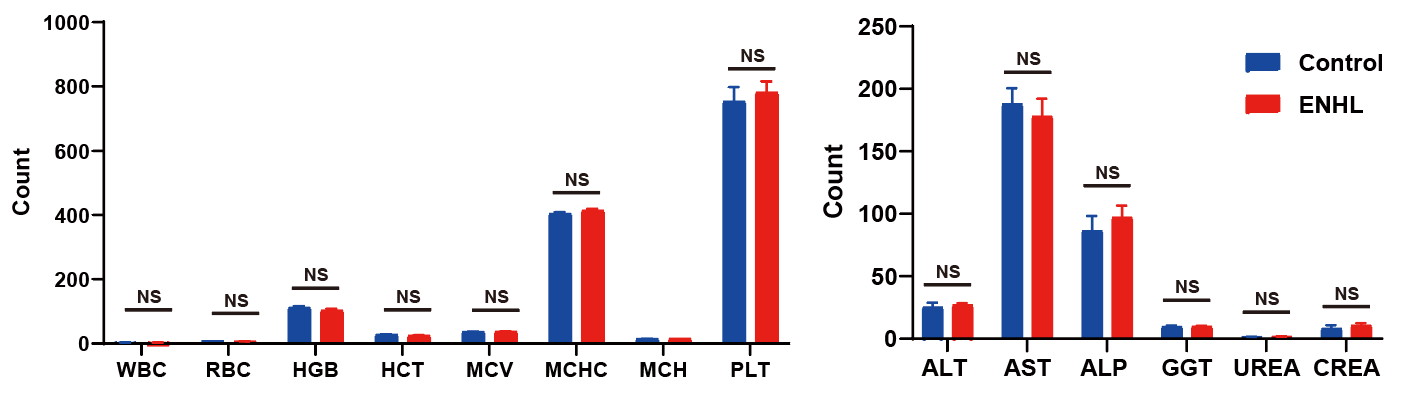


**Figure S25.** Biosafety evaluation. Complete blood count parameters analysis of mice (n = 5, left) and serum biochemical markers of liver and kidney function (Mean ± SD, n = 5, right). Statistical significance was determined by one-way ANOVA with Tukey’s multiple comparisons (NS, not significant, *P* ＞ 0.05).


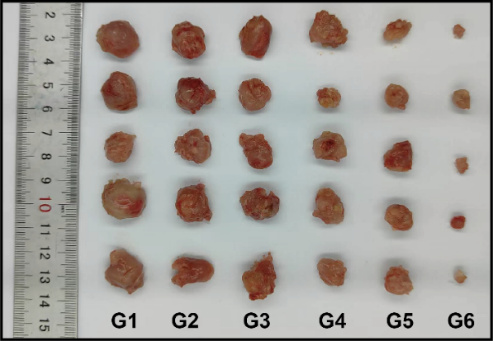


**Figure S26.** Gross morphological assessment of tumor dimensions in mouse models with low rectal cancer following different treatment regimens (PBS gavage (G1), ENHP gavage (G2), ENHL gavage (G3), 4 Gy (G4), ENHP gavage + 4 Gy (G5), ENHL gavage + 4 Gy (G6); n = 5).


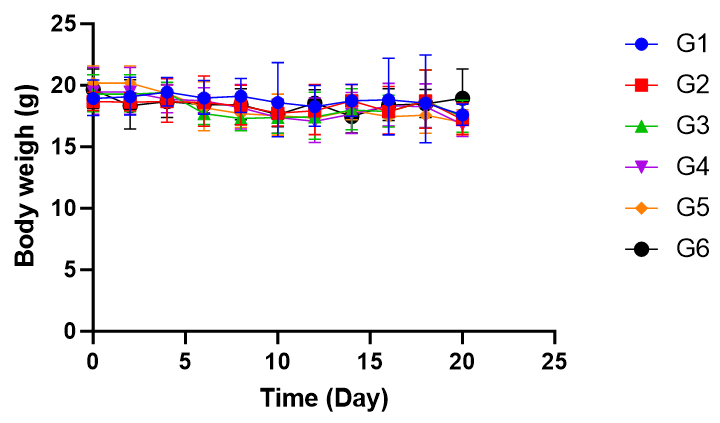


**Figure S27.** Body weight measurements of mice in different treatment groups (PBS gavage (G1), ENHP gavage (G2), ENHL gavage (G3), 4 Gy (G4), ENHP gavage + 4 Gy (G5), ENHL gavage + 4 Gy (G6). n = 5). Statistical significance was determined by one-way ANOVA with Tukey’s multiple comparisons (**P* < 0.05, ***P* < 0.01, ****P* < 0.001, *****P* < 0.0001).


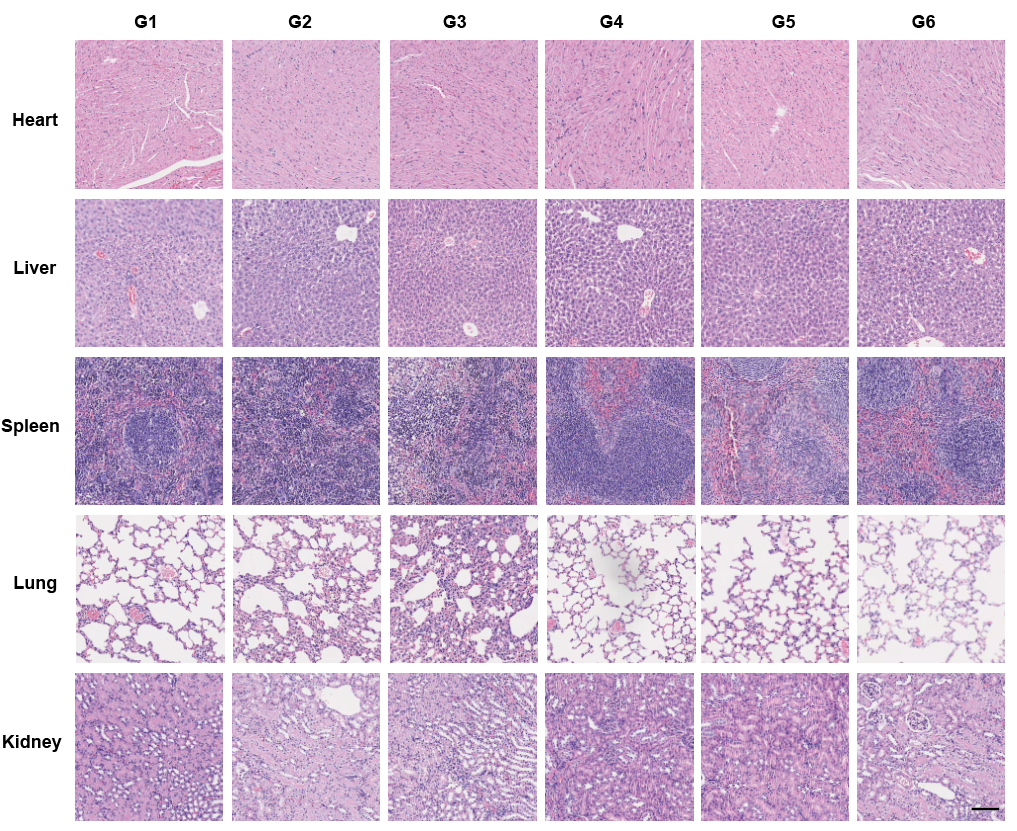


**Figure S28.** Representative H&E-stained images of major organs from mice after different treatments (PBS gavage (G1), ENHP gavage (G2), ENHL gavage (G3), 4 Gy (G4), ENHP gavage + 4 Gy (G5), ENHL gavage + 4 Gy (G6). n = 5). Scale bar, 100 μm.


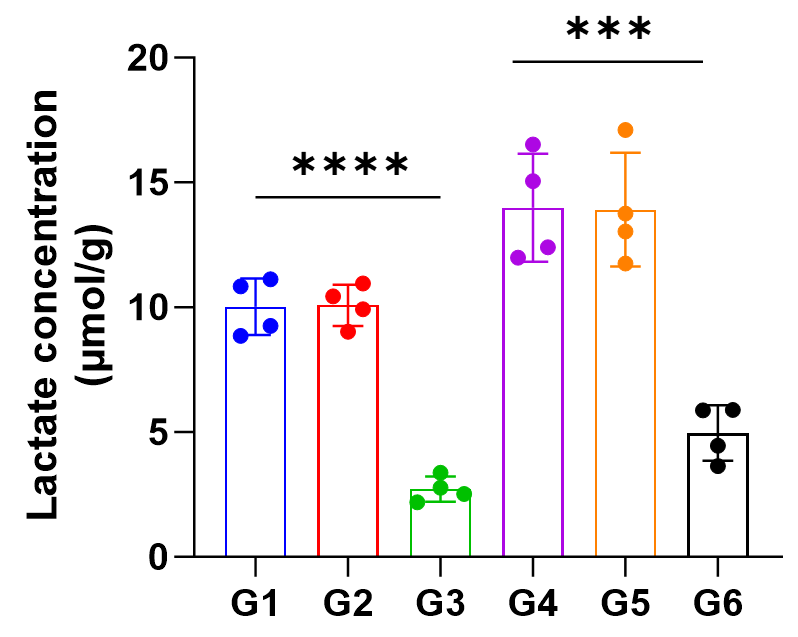


**Figure S29.** The lactate levels in CT26 orthotopic tumors after different treatments (PBS gavage (G1), ENHP gavage (G2), ENHL gavage (G3), 4 Gy (G4), ENHP gavage + 4 Gy (G5), ENHL gavage + 4 Gy (G6). Mean ± SD, n = 4). Statistical significance was determined by one-way ANOVA with Tukey’s multiple comparisons (**P* < 0.05, ***P* < 0.01, ****P* < 0.001, *****P* < 0.0001).


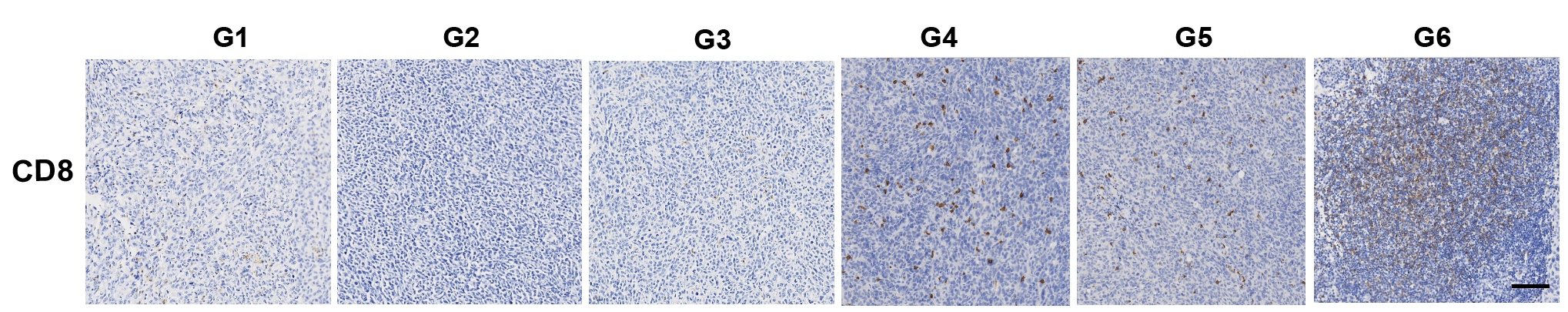


**Figure S30.** Representative images of CD8 staining of tumor tissues from mice after different treatments (PBS gavage (G1), ENHP gavage (G2), ENHL gavage (G3), 4 Gy (G4), ENHP gavage + 4 Gy (G5), ENHL gavage + 4 Gy (G6). n = 5). Scale bar, 100 μm.


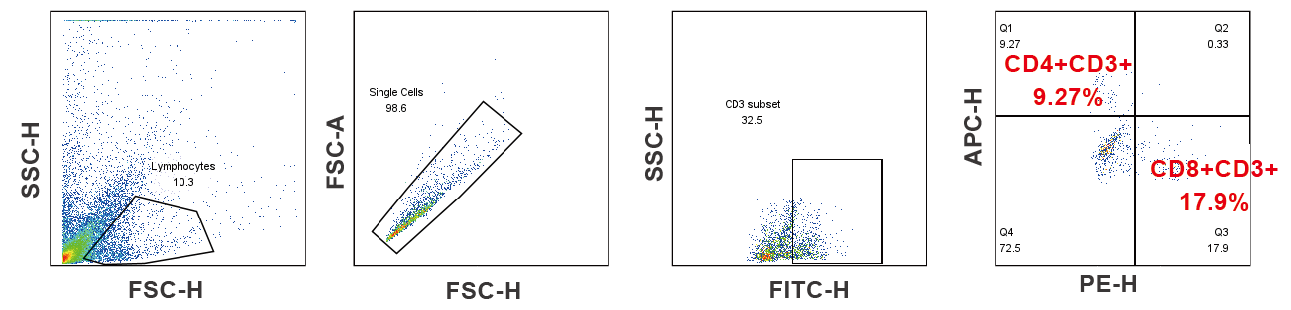


**Figure S31.** Gating strategy for flow cytometry (FCM) analysis of CD8^+^ T cells (CD45^+^CD3^+^CD8^+^).


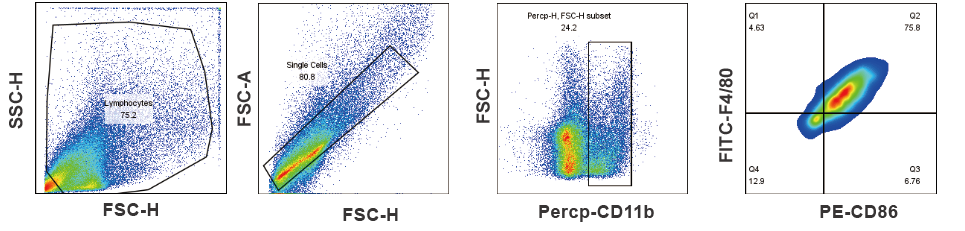


**Figure S32.** Gating strategy for FCM analysis of M1-like TAMs (CD11b^+^F4/80^+^CD86^+^).


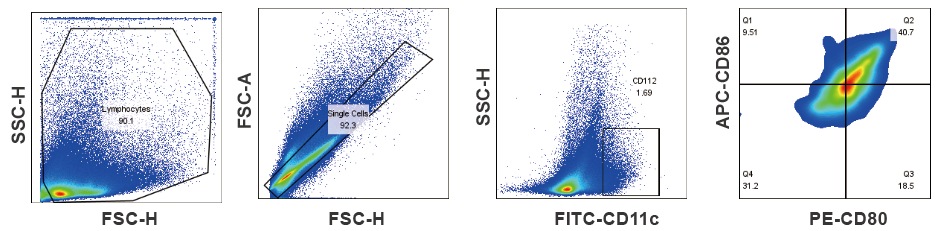


**Figure S33.** Gating strategy for FCM analysis of mature DCs (CD11c^+^ CD80^+^ CD86^+^).


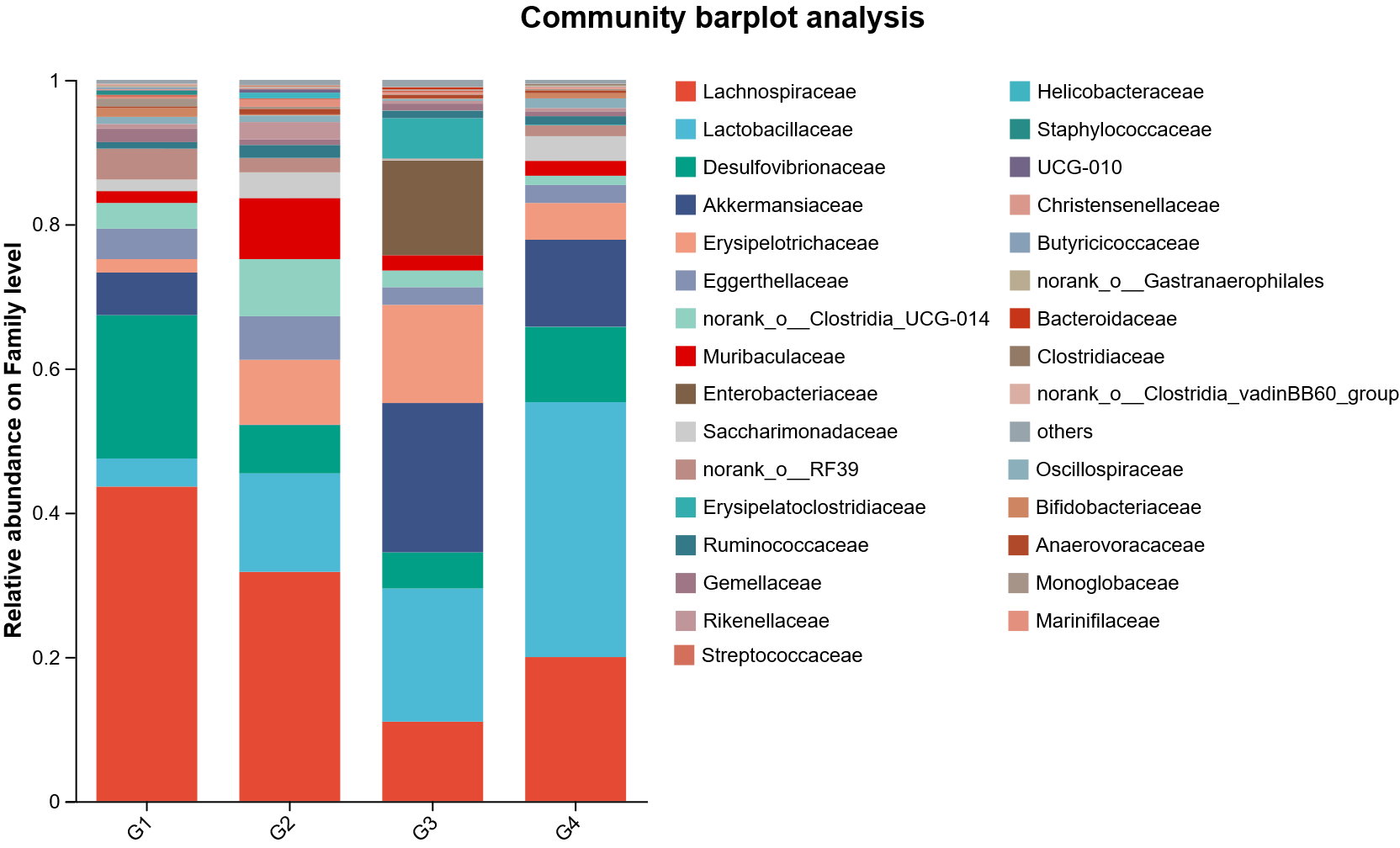


**Figure S34.** Relative abundance of gut microbiota at the Family level (G1: PBS control; G2: ENHL; G3: 4 Gy; G4: ENHL + 4 Gy).


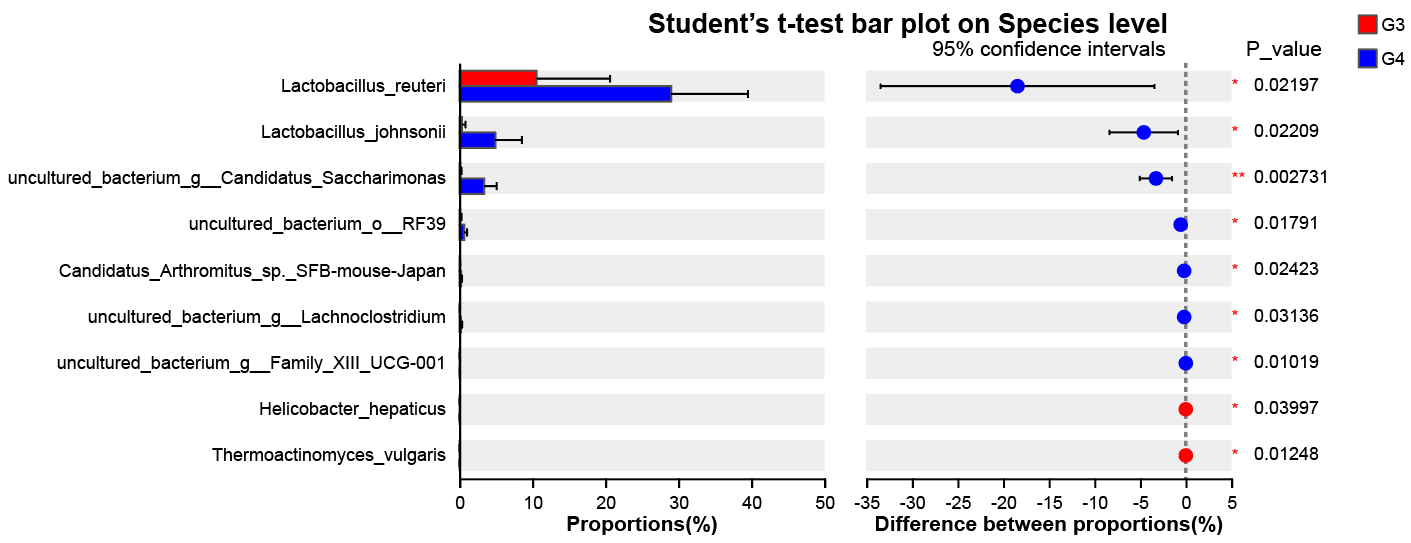


**Figure S35.** Relative abundance of gut microbiota at the Species level (G1: PBS control; G2: ENHL; G3: 4 Gy; G4: ENHL + 4 Gy).


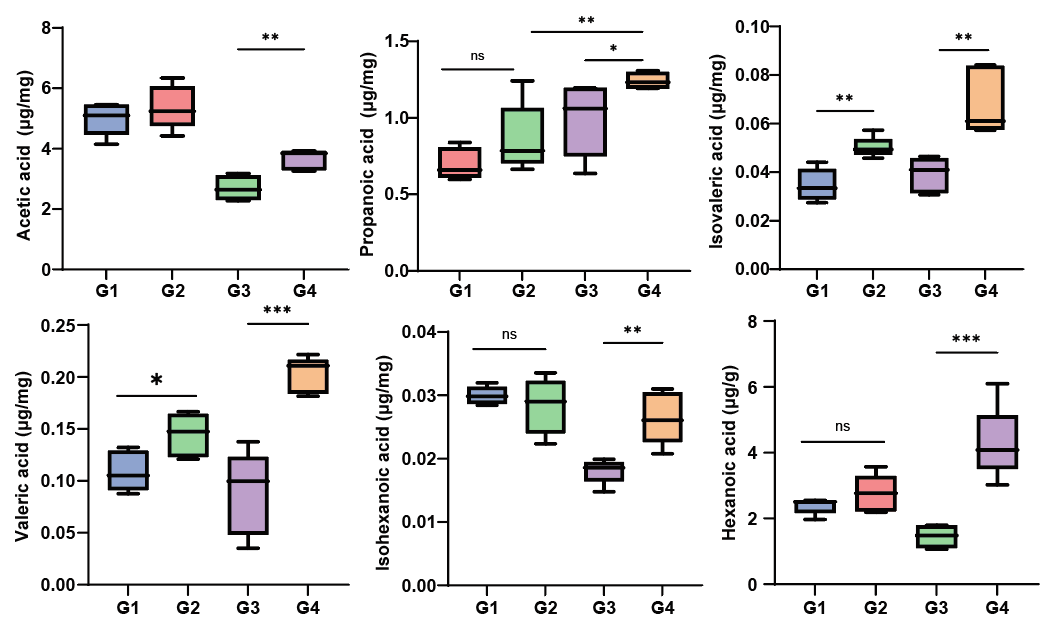


**Figure S36.** Statistical analysis of short-chain fatty acid, including acetic acid, propanoic acid, isobutyric acid, valeric acid, isohexanoic acid, and hexanoic acid (G1: PBS control; G2: ENHL; G3: 4 Gy; G4: ENHL + 4 Gy; Mean ± SD, n = 5). Statistical significance was determined by one-way ANOVA with Tukey’s multiple comparisons (**P* < 0.05, ***P* < 0.01, ****P* < 0.001, *****P* < 0.0001, ns. not significant).


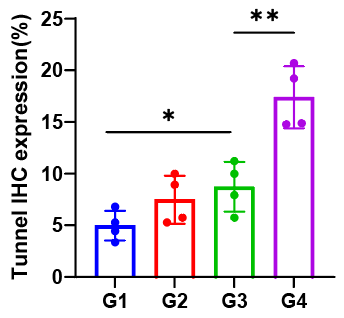


**Figure S37.** Quantitative analysis of Tunnel-positive cells (G1: PBS control; G2: ENHL; G3: 4 Gy; G4: ENHL + 4 Gy; Mean ± SD, n = 4). Statistical significance was determined by one-way ANOVA with Tukey’s multiple comparisons (**P* < 0.05, ***P* < 0.01, ****P* < 0.001, *****P* < 0.0001).


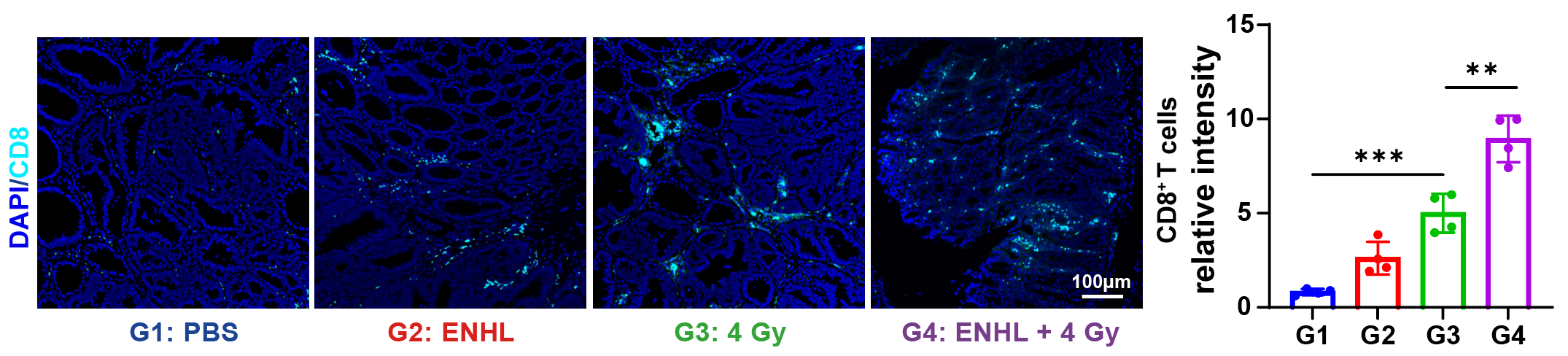


**Figure S38.** Immunofluorescence staining images and relative intensity of FITC channel within CD8-positive regions of interest in different treatment groups (G1: PBS control; G2: ENHL; G3: 4 Gy; G4: ENHL + 4 Gy; Mean ± SD, n = 4). Statistical significance was determined by one-way ANOVA with Tukey’s multiple comparisons (**P* < 0.05, ***P* < 0.01, ****P* < 0.001, *****P* < 0.0001).


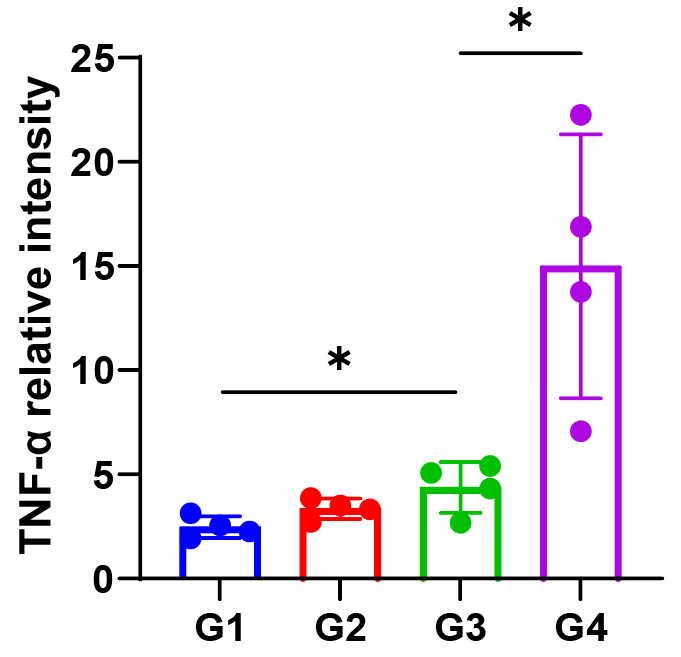


**Figure S39**. Relative intensity of TNF-α in different treatment groups (G1: PBS control; G2: ENHL; G3: 4 Gy; G4: ENHL + 4 Gy; Mean ± SD, n = 4). Statistical significance was determined by one-way ANOVA with Tukey’s multiple comparisons (**P* < 0.05, ***P* < 0.01, ****P* < 0.001, *****P* < 0.0001).

**Table S1**. **Clinical information**

| **Group** | **TRG** | **Sex** | **Age** | **Tumor size (cm)** | **Differentiation, degree** | **Lymph node metastasis** |
| --- | --- | --- | --- | --- | --- | --- |
| **Resistance** | **3** | **0** | **75** | **4.5**×**3.5**×**1.5** | **1** | **1** |
|  | **3** | **1** | **55** | **1.8×0.9×0.8** | **2** | **0** |
|  | **3** | **1** | **54** | **1.8×0.9×0.8** | **2** | **0** |
|  | **3** | **0** | **67** | **1.5** | **1** | **0** |
|  | **3** | **0** | **71** | **5**×**4**×**1** | **1** | **0** |
|  | **3** | **0** | **87** | **7.5**×**6.5**×**3** | **1** | **2** |
|  | **2** | **1** | **51** | **3.5**×**2**×**0.8** | **1** | **0** |
|  | **2** | **0** | **66** | **1.2**×**1.2**×**0.5** | **1** | **0** |
|  | **2** | **1** | **45** | **5.5**×**2**×**1** | **1** | **0** |
|  | **2** | **1** | **56** | **3**×**1.5**×**1** | **1** | **1** |
|  | **2** | **0** | **57** | **2.5**×**2.5**×**1.2** | **1** | **0** |
|  | **2** | **1** | **63** | **2.2**×**2**×**0.8** | **3** | **0** |
| **Sensitive** | **1** | **0** | **67** | **0.8×0.6×0.5** | **3** | **0** |
|  | **1** | **0** | **66** | **1**×**1**×**1** | **3** | **1** |
|  | **1** | **1** | **33** | **4**×**3.5**×**1.2** | **1** | **0** |
|  | **1** | **0** | **64** | **2×1.2** | **3** | **0** |
|  | **1** | **0** | **34** | **0.8×0.7** | **3** | **1** |
|  | **1** | **0** | **67** | **3**×**2.5**×**1** | **3** | **2** |

**Table S1.** The Mandard Tumor Regression Grade (TRG) system was utilized to evaluate the efficacy of neoadjuvant radio-chemotherapy: TRG0 (complete pathological response) and TRG1 (marked regression) were classified as the sensitive group, while TRG2 (partial regression) and TRG3 (minimal or no regression) were categorized as the resistant group. Variable definitions were as follows: gender (0=male, 1=female); tumor differentiation grade (0=well-differentiated, 1=moderately differentiated, 2=poorly differentiated, 3=special subtype); lymph node metastasis status (0=no metastasis, 1=1-3 metastatic nodes [N1], 2=4-6 metastatic nodes [N2]).

**Table S2**. **Plasmid information.**

| **1. Myc-EGFP**  CTCGAGATGGTCAGCAAAGCCAAGAAAACATCCATTACAAAACCTTTACCGTGGAAGGCGAAGGCGAAGGCAACAGCCATGAAGGCCAACGCGCTGGGCACCACCTTTGGCTATGGCATGAAATATTATACCAAATATCCGAGCGGCCTGAAAAACTGGTTCGCGAAGTGATCCGGCGGITTACCTATGATCGCCATATTCAGTATAAAGGCGATGGCAGCATTCATGCGAAACATCAGCATTTTATGAAAAACGGCACCTATCATAACATGTCCAATTACCGGCCAAGATAAAGAAAACACCCACACCCGCCATGAGGCGGGAATGCCCGGTGACCCTGCGTATCCCTCTACCATTACGCATCAGTATACCATTGCAAACCGCTGCATAATCAGCCGGCGCCGGATGTGCCGTATCATTGGATCGCAAACAGTATACGCAGAGCAAAGATGATGCGGAAGAACCCGATCATATTTGTCAGAGCGAAACCCTGGAAGCGCATCTGAAAGGCATGGATGAACTGTATAAATGAGAATTC |
| --- |
| **2. MyC-INP-HIpA-RBS-ClyA-EGFP****-3×Flag**  GCGGCCGCAATGGTGAGCAAAGGCGAAGAGGAAAACATGGCGAGCACCCCGTTTAAATTTCAGCTGAAAGGCACCATTAACGGCAAAAGCTTTACCGTGGAAGGCGAAGGCGAAGGCAACAGCCATGAAGGCAGCCATAAAGGCAAATATGTGTGCACGAGCGGCAAACTGCCGATGAGCTGGGCGGCGCTGGGCACCACCTTTGGCTATGGCATGAAATATTATACCAAATATCCGAGCGGCCTGAAAAACTGGTTTCGCGAAGTGATGCCGGGCGGCTTTACCTATGATCGCCATATTCAGTATAAAGGCGATGGCAGCATTCATGCGAAACATCAGCATTTTATGAAAAACGGCACCTATCATAACATTGTGGAATTTACCGGCCAAGATTTTAAAGAAAACAGCCCGGTGCTGACCGGCGATATGAACGTGAGCCTGCCGAACGAAGTGCCGCAGATTCCGCGCGATGATGGCGTGGAATGCCCGGTGACCCTGCTGTATCCGCTGCTGAGCGATAAAAGCAAATATGTTGAAGCGCATCAGTATACCATTTGCAAACCGCTGCATAATCAGCCGGCGCCGGATGTGCCGTATCATTGGATTCGCAAACAGTATACGCAGAGCAAAGATGATGCGGAAGAACGCGATCATATTTGTCAGAGCGAAACCCTGGAAGCGCATCTGAAAGGCATGGATGAACTGTATAAAGGCGCCGGCG |
| **3. MyC-INP-HIpA-ClyA-LOx-3×Flag**  GAACAAAAACTCATCTCAGAAGAGGATCTGCTCGAGATGACCCTGGACAAAGCCCTGGTTCTGCGCACTTGTGCAAACAACATGGCCGATCACTGCGGTCTTATCTGGCCAGCGTCCGGTACTGTGGAATCTCGTTACTGGCAGTCTACCCGTCGCCATGAGAACGGTCTGGTAGGTTTACTGTGGGGTGCTGGCACCAGCGCATTCCTCAGCGTGCATGCAGATGCTCGTTGGATTGTCTGTGAAGTTGCCGTTGCAGACATCATCAGTCTGGAAGAGCCGGGTATGGTGAAGTTTCCGCGTGCCGAAGTGGTTCATGTCGGCGACCGTATTAGCGCGTCTCACTTCATTTCGGCACGTCAGGCCGACCCTGCGTCTACCTCAACTTCTACGTCCACGAGTACGCTGACTCCAATGCCTACGGCCATTCCGACGCCAATGCCTGCGGTTGCAAGCGTAACGCTGCCAGTGGCAGAACAGGCACGTCATGAAGTGTTTGATGTAGCGTCGGTAAGCGCTGCTGCCGCACCAGTCAACACTCTGCCAGTTACTACGCCGCAGAATCTGCAGACCGCAACTTATGGTTCTACGTTGTCCGGCGACAATCATTCTCGTCTGATTGCAGGTTATGGTTCCAACGAGACCGCTGGCAACCACAGTGATCTGATTGGGTCCTTTGGTACCATGGCGAACAAACAGGATCTGATTGCGAAAGTGGCGGAAGCGACCGAACTGACCAAAAAAGATAGCGCGGCGGCGGTGGATGCGGTGTTTAGCGCGATTGAAAGTTTTCTGAGCGAAGGCGAAAAAGTGCAGCTGATTGGCTTTGGCAACTTTGAAGTGCGCGAACGCGCGGCGCGCAAAGGCCGCAACCCGCAGACCGGCGCGGAAATTAAAATTGCGGCGAGCAAAGTGCCGGCGTTTAAAGCGGGCAAAGCGCTGAAAGATGCGGTGAAATAAAAAGAGGAGAAAGAATTATGGGCATGACTGAAATCGTCGCGGATAAAACCGTTGAGGTGGTGAAAAACGCAATCGAAACCGCTGATGGCGCTCTGGATCTGTATAACAAATACCTGGACCAGGTTATTCCGTGGCAGACTTTTGATGAAACCATTAAAGAACTGTCCCGCTTCAAACAGGAATACTCTCAGGCGGCGTCCGTACTGGTAGGCGACATCAAAACTCTGCTGATGGACTCCCAGGACAAATACTTCGAAGCCACTCAGACCGTTTACGAGTGGTGCGGTGTTGCTACCCAGCTGCTGGCTGCATACATTCTGCTGTTCGACGAATATAATGAGAAAAAAGCGTCTGCTCAAAAAGACATCCTGATCAAAGTTCTGGATGATGGTATCACTAAGCTGAACGAAGCGCAGAAAAGCCTGCTGGTTAGCAGCCAGAGCTTCAACAACGCGTCTGGTAAGCTGCTGGCTCTGGATTCCCAGCTGACCAACGATTTCTCCGAAAAATCTTCCTACTTCCAGTCCCAGGTAGACAAAATCCGCAAAGAAGCTTACGCTGGCGCAGCTGCTGGTGTTGTAGCAGGTCCGTTCGGTCTGATCATTAGCTATTCTATTGCTGCAGGTGTGGTCGAGGGCAAACTGATTCCGGAGCTGAAAAACAAACTGAAATCTGTACAAAACTTCTTTACCACCCTGTCTAACACTGTTAAACAGGCGAACAAAGACATCGATGCGGCAAAACTGAAACTGACCACCGAGATCGCGGCCATCGGTGAAATCAAAACTGAAACCGAAACCACCCGTTTTTACGTTGATTACGACGACCTGATGCTGAGCCTGCTGAAAGAGGCAGCGAAAAAAATGATCAACACTTGTAACGAATACCAGAAACGTCACGGTAAAAAAACCCTGTTTGAAGTGCCGGAAGTAGGCGGTGGCGGCAGTGCGGCCGCAAACAATAACGATATTGAATATAACGCGCCGAGCGAAATTAAATATATTGATGTGGTGAACACCTATGATCTGGAAGAGGAAGCGAGCAAAGTGGTGCCGCATGGCGGCTTTAACTATATTGCGGGCGCGAGCGGTGATGAATGGACCAAACGCGCGAACGATCGCGCGTGGAAACATAAACTGCTGTATCCGCGCCTGGCGCAAGATGTGGAAGCGCCGGATACGAGCACCGAAATTCTGGGCCATAAAATTAAAGCGCCGTTTATTATGGCGCCGATTGCGGCGCATGGCCTGGCGCATACCACCAAAGAAGCGGGCACCGCGCGCGCGGTGAGCGAATTTGGCACCATTATGAGCATTAGCGCCTACAGCGGCGCGACCTTTGAAGAAATTAGCGAAGGCCTGAACGGCGGCCCGCGCTGGTTTCAGATTTATATGGCGAAAGATGATCAGCAGAACCGCGATATTCTGGATGAAGCGAAAAGCGATGGCGCGACCGCGATTATTCTGACCGCGGATAGCACCGTGAGCGGCAACCGCGATCGCGATGTGAAAAACAAATTTGTGTATCCGTTTGGCATGCCGATTGTGCAGCGCTATCTGCGCGGCACCGCGGAAGGCATGAGCCTGAACAACATTTATGGCGCGAGCAAACAGAAAATTAGCCCGCGCGATATTGAAGAAATTGCGGGCCATAGCGGCCTGCCGGTGTTTGTGAAAGGCATTCAGCATCCGGAAGATGCGGATATGGCGATTAAACGCGGTGCGAGCGGCATTTGGGTGAGCAACCATGGCGCGCGTCAGCTGTATGAAGCGCCGGGCAGCTTTGATACCCTGCCGGCGATTGCGGAACGCGTGAACAAACGCGTGCCGATTGTGTTTGATAGCGGCGTGCGCCGCGGCGAACATGTGGCGAAAGCGCTGGCGAGCGGCGCGGATGTTGTTGCGCTGGGTCGCCCGGTTCTGTTTGGTCTGGCGCTGGGCGGCTGGCAAGGCGCGTATAGTGTGCTGGATTATTTTCAGAAAGATCTGACCCGCGTGATGCAGCTGACCGGCAGTCAGAACGTGGAAGATCTGAAAGGCCTGGATCTGTTTGATAACCCGTATGGCTATGAATATGGCGCCGGCGCAGACTACAAAGACCATGACGGTGATTATAAAGATCATGACATCGACTACAAGGATGACGATGACAAGTAAGAATTC |
